# Alternative splicing promotes tumour aggressiveness and drug resistance in African American prostate cancer

**DOI:** 10.1038/ncomms15921

**Published:** 2017-06-30

**Authors:** Bi-Dar Wang, Kristin Ceniccola, SuJin Hwang, Ramez Andrawis, Anelia Horvath, Jennifer A. Freedman, Jacqueline Olender, Stefan Knapp, Travers Ching, Lana Garmire, Vyomesh Patel, Mariano A. Garcia-Blanco, Steven R. Patierno, Norman H. Lee

**Affiliations:** 1 Department of Pharmacology and Physiology, School of Medicine and Health Sciences, The George Washington University, Washington, District Of Columbia 20037, USA; 2 Department of Pharmaceutical Sciences, School of Pharmacy and Health Professions, University of Maryland Eastern Shore, Princess Anne, Maryland 21853, USA; 3 Department of Microbiology, Immunology and Tropical Medicine, School of Medicine and Health Sciences, The George Washington University, Washington, District Of Columbia 20037, USA; 4 Department of Urology, School of Medicine and Health Sciences, The George Washington University, Washington, District Of Columbia 20037, USA; 5 Duke Cancer Institute and Department of Medicine, Duke University Medical Center, Durham, North Carolina 27710, USA; 6 Department of Clinical Pharmacology, University of Oxford, Oxford OX3 7BN, UK; 7 The Nuffield Department of Clinical Medicine, Structural Genomics Consortium, University of Oxford, Oxford OX3 7BN, UK; 8 Cancer Epidemiology Program, University of Hawaii Cancer Center, Honolulu, Hawaii 96813, USA; 9 Oral and Pharyngeal Cancer Branch, National Institute of Dental and Craniofacial Research, National Institutes of Health, Bethesda, Maryland 20892, USA; 10 Department of Biochemistry & Molecular Biology, The University of Texas Medical Branch at Galveston, Galveston, Texas 77555, USA

## Abstract

Clinical challenges exist in reducing prostate cancer (PCa) disparities. The RNA splicing landscape of PCa across racial populations has not been fully explored as a potential molecular mechanism contributing to race-related tumour aggressiveness. Here, we identify novel genome-wide, race-specific RNA splicing events as critical drivers of PCa aggressiveness and therapeutic resistance in African American (AA) men. AA-enriched splice variants of *PIK3CD*, *FGFR3*, *TSC2* and *RASGRP2* contribute to greater oncogenic potential compared with corresponding European American (EA)-expressing variants. Ectopic overexpression of the newly cloned AA-enriched variant, *PIK3CD-S*, in EA PCa cell lines enhances AKT/mTOR signalling and increases proliferative and invasive capacity *in vitro* and confers resistance to selective PI3Kδ inhibitor, CAL-101 (idelalisib), in mouse xenograft models. High *PIK3CD-S* expression in PCa specimens associates with poor survival. These results highlight the potential of RNA splice variants to serve as novel biomarkers and molecular targets for developmental therapeutics in aggressive PCa.

## Abstract (web summary)

In prostate cancer tumour aggressiveness can be related to race. Here, the authors identify an alternative RNA splice variant of PIK3CD as a potential mechanism to explain racial disparities in the incidence and aggressiveness of prostate cancer between African American and European American men.

Prostate cancer (PCa) is the most commonly diagnosed cancer and the second leading cause of cancer death among American men^1^. Striking population disparities in PCa risk and clinical outcome have been observed across racial groups. Notably, African American (AA) men exhibit 1.6-fold higher incidence and 2.4-fold higher mortality rates of PCa compared with European American (EA) men^2^,^3^. Socioeconomic factors remain a major component accounting for the PCa disparities between AA and EA populations^3^,^4^. However, higher mortality and recurrence rates are still observed in AA PCa even after adjustment of socioeconomic factors^5^, suggesting that intrinsic biological differences also play a contributing role in PCa disparities^6^,^7^,^8^,^9^,^10^.

Alternative splicing (AS) is a post-transcriptional process allowing for the generation of alternative mRNA transcripts that encode structurally and functionally disparate protein isoforms. Next-generation sequencing suggests that >90% of human genes undergo AS^11^, and the resulting complexity in the transcriptome explains how ∼20,000 protein-coding genes in the genome can lead to >250,000 distinct proteins in the proteome. Accumulating evidence indicates that alternative and/or aberrant splicing of precursor (pre)-mRNA plays an important but largely underappreciated role in cancers^12^,^13^,^14^,^15^, including PCa^16^. For example, the B-cell lymphoma 2-like 1 (*BCL2L1*) pre-mRNA is alternatively spliced into two variants, *Bcl-xS* and *Bcl-xL*, encoding protein isoforms with opposite biological effects^17^. Bcl-xS is a pro-apoptotic protein, while BcL-xL has anti-apoptotic properties conferring chemotherapy resistance in PCa cell line PC-3 (ref. 15) as well as castration-resistant xenograft growth^18^. Manipulation of splicing to decrease Bcl-xL and increase Bcl-xS levels has been shown to reduce tumour load^19^. Fibroblast growth factor receptor 2 (*FGFR2*) pre-mRNA also undergoes AS, where *FGFR2-IIIb* is predominately expressed in epithelial cells and *FGFR2-IIIc* is primarily associated with epithelial-to-mesenchymal transition of PCa cells^20^. Another example is the *TMPRSS2-ERG* gene fusion commonly found in PCa and associated with poor clinical outcome^21^,^22^. In a comparative study of *TMPRSS2-ERG* variants ectopically overexpressed in prostatic epithelial cells, variants containing a 72 base exon (*+72*) mediate increased cell proliferation and invasion^14^. Androgen receptor (AR) signalling is critically associated with PCa growth^23^ and splice variant *AR-V7* is overexpressed in hormone-refractory PCa, being correlated with poor patient survival and higher recurrence rates^24^,^25^.

Despite the significance of alternative/aberrant splicing in PCa progression irrespective of race, the occurrence of race-specific/-enriched PCa splicing events and a causal relationship between these events and observed PCa disparities remains unexplored. For example, it is unclear whether *FGFR2-IIIc*, *TMPRSS2-ERG+72*, *AR-V7* and/or other as yet undiscovered variants associated with more aggressive PCa might be predominantly or selectively expressed in AA PCa, thus contributing to PCa disparities. In addition, it is unknown whether differences in mRNA splicing along racial/population lines occur in only a limited number of genes or more globally across the transcriptome. If the latter, it will be important to ascertain whether these genome-wide, differential splicing (DS) events are overrepresented within specific gene ontologies (that is, proto-oncogenes, tumour suppressor genes). Lastly, assessment of the functional consequences of any race-specific (or enriched) splicing events will provide critical further insight into the genetic/molecular mechanisms underlying PCa disparities. To this end, we have applied a functional genomics approach to address these questions. Our results underscore the leveraging of population differences in tumour biology to discover novel splice variants that will likely serve as novel biomarkers and/or molecular targets for developmental therapeutics against aggressive AA PCa, identify previously hidden splice variants encoding oncogenic signalling proteins resistant to small-molecule inhibitors (SMIs), and assimilate splice variant information for prognostication of cancer aggressiveness and/or therapeutic responsiveness.

## Results

### Genome-wide DS events in AA versus EA PCa

**Figure 1:**
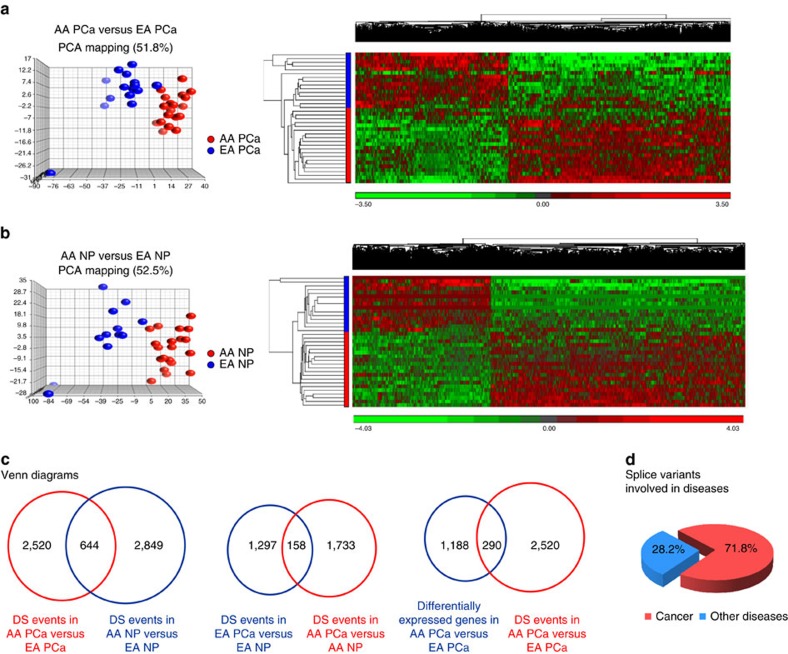
Differential alternative splicing events in AA PCa compared with EA PCa and AA NP compared with EA NP specimens. (**a**) Principal component analysis (PCA) plot and two-dimensional (2D) clustergram depicting 3,112 significant differentially expressed exons in 20 independent AA PCa versus 15 independent EA PCa specimens. (**b**) PCA plot and 2D clustergram depicting 3,384 significant differentially expressed exons in 20 AA NP versus 15 EA NP specimens. AA and EA specimens are represented by red and blue circles/bars, respectively. Rows represent specimens and columns represent exons in hierarchical clustergrams. Log2 expression values of exons were subjected to 2D unsupervised hierarchical clustering using average linkage method and Euclidean distance. (**c**) Venn diagrams of DS events in AA PCa versus EA PCa and AA NP versus EA NP, DS events in EA PCa versus EA NP and AA PCa versus AA NP and differentially expressed genes in AA PCa versus EA PCa and DS events in AA PCa versus EA PCa. (**d**) A majority of the genes with DS events in AA PCa versus EA PCa were functionally associated with cancer. The top three ‘other diseases’ were gastrointestinal disease, organismal injury and abnormalities and reproductive system disease.

A total of 35 PCa (20 AA/15 EA) and 35 patient-matched normal prostate (NP) specimens (20 AA/15 EA) derived from chemo-/hormone-/radiation-naive patients were interrogated using the Affymetrix Human Exon 1.0 ST GeneChip to assess DS events. Gleason scores of PCa specimens (range 6–8) and patient ages (range 49–81 years) were not significantly different between AA and EA cohorts (*P*>0.05, Fisher’s exact test). In AA PCa versus EA PCa and AA NP versus EA NP, the significant differentially expressed exons (Fig. 1a,b) could be modelled using the AS analysis of variance (ANOVA) approach^26^ into 2,520 and 2,849 DS events, respectively (Supplementary Data 1 and Supplementary Fig. 1). As depicted in the Venn diagram (Fig. 1c), 1,876 genes (2,520 minus 644) exhibited DS events unique to AA PCa versus EA PCa, 2,205 differentially spliced genes (2,849-644) were unique to AA NP versus EA NP and 644 DS events were in common (that is, DS events preexisting in AA NP versus EA NP and preserved in AA PCa versus EA PCa). Examples of genes with preexisting DS events included *PIK3CD*, *ITGA4* and *MET*, while *RASGRP2*, *NF1* and *BAK1* are examples of differentially spliced genes occurring only in AA PCa versus EA PCa. In EA PCa versus EA NP and AA PCa versus AA NP, the significant differentially expressed exons (Supplementary Fig. 2) could be modelled into 1,297 and 1,733 DS events, respectively (Fig. 1c). Presumably, a subset of 1,575 genes (1,733−158) with DS events unique to AA PCa may contribute to PCa disparities. Examples in this category included *FGFR3* and *TSC2* (Supplementary Data 1). On the other hand, a subset of 158 genes with DS events in common to both AA and EA PCa may contribute to PCa progression regardless of race (Fig. 1c). Consistently, such genes included *TMPRSS2* and *AR* (Supplementary Data 1c,d). Analysis of the exon array data employing both gene-wise^9^,^10^ and AS ANOVA modelling approaches^26^ identified 898 genes (1,188−290) that were differentially expressed but not exhibiting DS in AA PCa versus EA PCa, and 2,230 (2,520−290) genes undergoing DS but not differential expression (for example, level of variant ‘A’ for gene ‘X’ in AA PCa equivalent to variant ‘B’ for gene ‘X’ in EA PCa; Fig. 1c).

### Prevalence of DS events in cancer-associated pathways

We categorized genes undergoing DS in AA PCa versus EA PCa based on molecular function, gene ontology and disease association. Relevant cancer-related ontologies included cell growth and proliferation, cell death and survival, cellular movement, cell adhesion and DNA damage/repair (*P* values ranged from 6.54 × 10^−12^ to 1.88 × 10^−2^, Fisher’s exact test; Supplementary Data 2). Notably, a large fraction (1,816 out of 2,520, 71.8%) of the differentially spliced genes were discovered to be overrepresented across multiple cancers, including colorectal, renal, breast, brain, lung, stomach, prostate and haematologic cancers (*P* values ranged from 1.43 × 10^−9^ to 1.96 × 10^−2^, Fisher’s exact test; Fig. 1d and Supplementary Data 2). There was an unexpected skewing in the distribution of in-frame versus out-of-frame exon skipping events in cancer-related genes, where in-frame events were significantly favoured in AA over EA PCa specimens (*P*<0.05, Fisher’s exact test; Supplementary Table 1). This finding was in line with an overall significant preference for in-frame events across all genes (cancer-related and noncancer-related) in AA PCa specimens (Supplementary Table 1). In the case of noncancer-related genes only, there was no significant skewing of in-frame distribution events between AA versus EA PCa (Supplementary Table 1).

**Figure 2:**
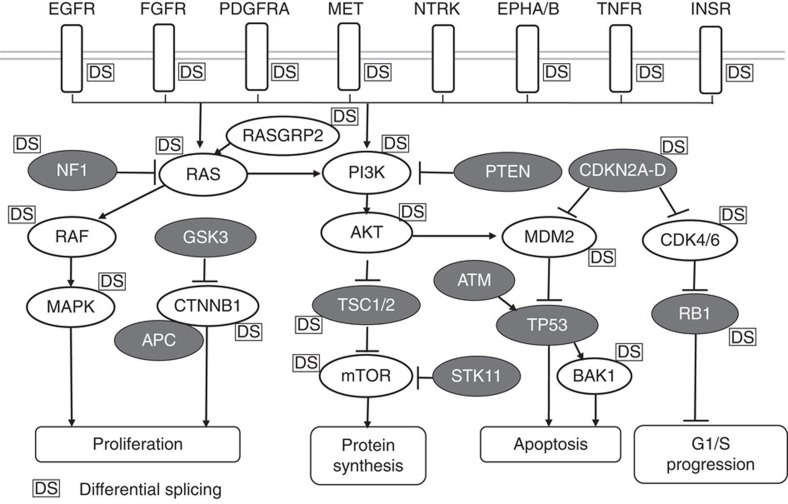
Representation of differential splicing events in a composite oncogenic signalling pathway. DS events in AA PCa versus EA PCa were frequently detected in multiple oncogenic signalling pathways. Depicted pathway is a composite of PI3K/AKT/mTOR, RAS/RAF/MAPK, CDK/RB1, MDM2/TP53 and WNT/GSK3/CTNNB1/APC signalling. Open figures indicate oncogenes and closed figures indicate tumour suppressor genes. ‘DS’ indicates that a differential alternative splicing event was detected for a particular oncogene or tumour suppressor gene.

We also examined the distribution of DS events across cell signalling pathways. There was a striking significant overrepresentation of DS events in multiple oncogenic signalling pathways, including epidermal growth factor (EGF), vascular endothelial growth factor (VEGF), phosphatase and tensin homolog (PTEN), phosphatidylinositol-3-kinase (PI3K)/AKT, extracellular signal–regulated kinase/mitogen-activated protein kinase (ERK/MAPK) and nuclear factor-κB (NF-κB) signalling (Fisher’s exact test, *P* values ranged from 0.00126 to 0.02089; Supplementary Fig. 3 and Supplementary Data 3). Interestingly, many of these same pathways are known to be mutated based on earlier cancer genome sequencing studies^27^,^28^,^29^. A composite oncogenic signalling pathway comprising DS events found in AA PCa versus EA PCa is depicted in Fig. 2. Taken together, our data provide strong evidence that DS events may play a critical role in PCa disparities.

### Validation of AS variants in AA versus EA PCa

**Figure 3:**
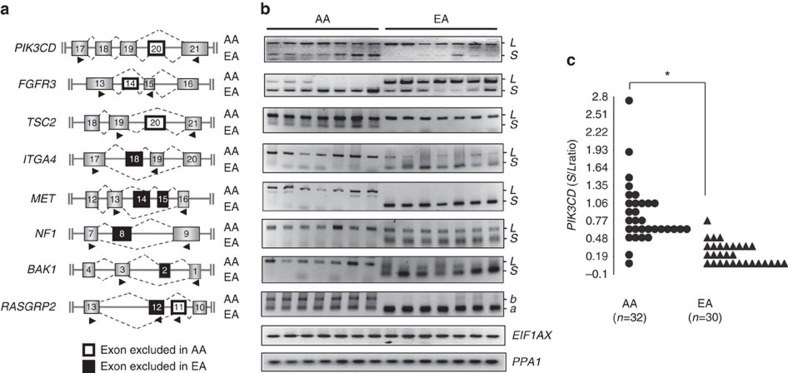
Validation of differential alternative splicing of oncogenes and tumour suppressor genes in AA versus EA PCa specimens. (**a**) Schematic representation of DS events within the indicated genes in AA PCa (top dashed lines connecting exons) and EA PCa (bottom dashed lines) based on alternative splicing ANOVA model. Closed arrowheads below exons represent primer location in qRT-PCR validation of alternatively spliced transcripts. (**b**) Representative RT-PCR results validating race-specific/-enriched variant transcripts in either AA PCa or EA PCa specimens. Shown are the RT-PCR results for the AA-specific/-enriched variants *PI3KCD-S*, *FGFR3-S*, *TSC2-S*, *ITGA4-L*, *MET-L*, *NF1-L*, *BAK1-L* and *RASGRP2-b*; and EA-specific/-enriched variants *PIK3CD-L*, *FGFR3-L*, *TSC2-L*, *ITGA4-S*, *MET-S*, *NF1-S*, *BAK1-S* and *RASGRP2-a*. Each lane represents an RT-PCR result from an independent PCa specimen that was also interrogated in exon array experiments. RT-PCR of *EIF1AX* and *PPA1* transcripts served as loading controls. The qRT-PCR results are summarized in Supplementary Fig. 4. Unprocessed RT-PCR images are shown in Supplementary Fig. 11. (**c**) Additional validation and quantification of the ratio of the AA-enriched *PI3KCD-S* (short) variant and the race-independent *PI3KCD-L* (long) variant in a separate cohort of PCa patient specimens. RNA was isolated from *n*=32 AA PCa and *n*=30 EA PCa specimens and subjected to qRT-PCR. Shown is a plot of the ratio of *S/L*. *EIF1AX* and *PPA1* transcripts served as internal normalization controls. *P<0.05 using two-sided Student’s *t*-test. Variance was similar among groups being compared.

We proceeded to validate a subset of both proto-oncogenes and tumour suppressor genes with DS events in our composite cancer signalling pathway, including *PIK3CD*, *FGFR3*, *TSC2*, *ITGA4*, *MET*, *NF1*, *BAK1*, *ATM* and *RASGRP2* (Fig. 2). Real-time PCR (RT-PCR) was performed on RNA samples obtained from AA and EA PCa specimens originally interrogated by the exon arrays. Primer pairs or trios were designed for RT-PCR to amplify simultaneously multiple variants of each gene (Fig. 3a). As shown in Fig. 3b, AA PCa specimens contained both *PIK3CD* long (*PIK3CD-L*, including exon 20) and short (*PIK3CD-S*, missing exon 20) variants, whereas EA PCa samples predominately expressed *PIK3CD-L*. Hence, the RT-PCR results were in agreement with the exon array data, indicating the presence of an AA-enriched *PIK3CD-S* variant. Analogous findings were obtained where either a short or long variant of each gene was confirmed by RT-PCR to be enriched or uniquely expressed in AA (*TSC2-S*, *ITGA4-L*, *MET-L*, *BAK1-L*) or EA PCa (*FGFR3-L*, *ITGA4-S*, *MET-S*, *NF1-S*, *BAK1-S*) (Fig. 3b; Supplementary Fig. 4 for quantitative RT-PCR (qRT-PCR) results from *n*=22–25 AA and *n*=21–24 EA PCa specimens). Exon array data also revealed two alternative *RASGRP2* transcripts with apparent mutually exclusive exon skipping events (Fig. 3a and Supplementary Fig. 1d). RT-PCR validation likewise confirmed that a *RASGRP2-b* variant (exon 11 excluded) was exclusively expressed in AA PCa, while a *RASGRP2-a* variant (exon 12 excluded) was enriched in EA PCa (Fig. 3b and Supplementary Fig. 4). We were unable to validate DS of *ATM*, whereas two additional genes (*GSK3A* and *EPHA1*) identified by exon arrays as not undergoing DS were confirmed by RT-PCR. In summary, there was strong agreement (10/11, 91%) between exon array and RT-PCR results, thus providing an internal quality metric to our global DS analysis of AA and EA PCa (see Methods for additional metrics).

The race-dependent expression of *PIK3CD* variants was particularly interesting owing to recent findings implicating PI3Kδ (p110δ) kinase activity in haematologic malignancies as well as other cancer types^30^,^31^,^32^,^33^. In a separate cohort of PCa specimens obtained from 32 AAs (age range 52–76 years, Gleason score range 6–8) and 30 EAs (age range 50–82 years, Gleason score range 6–8; not significantly different from AA, Fisher’s exact test, *P*>0.05), quantitative RT-PCR validation was performed reaffirming significantly higher levels of *PIK3CD-S* relative to *PIK3CD-L* in AA versus EA PCa specimens (Fig. 3c). Given the robustness and potential significance of these findings, subsequent *in vitro* and *in vivo* studies centred on the *PIK3CD* variants, as described below.

### Molecular cloning of *PIK3CD* splice variants

The AA-enriched *PIK3CD-S* variant has never before been described in the literature nor the UCSC (University of California, Santa Cruz) (genome.ucsc.edu) or Ensembl Genome Browser (www.ensembl.org). Consequently, we cloned the full-length versions of *PIK3CD-S* from AA PCa cell line MDA PCa 2b, and *PIK3CD-L* from MDA PCa 2b as well as EA PCa cell lines VCaP and LNCaP using standard molecular approaches (5′- and 3′-RACE (rapid amplification of cDNA ends)^34^). We likewise cloned matching *PIK3CD-S* and *PIK3CD-L* variants from PCa patient specimens. Supplementary Fig. 5 schematically depicts the full-length clones of *PIK3CD-L* (comprising a total of 24 exons) along with three different AA *PIK3CD-S* variants (variant excluding exon 8, exon 20 or both exons 8 and 20) and one AA large deletion variant of the *PIK3CD* gene. Interestingly, exclusion of exon 8 eliminates a 30-amino acid segment situated between the Ras-binding and C2 domains, while exclusion of exon 20 deletes a 56-amino acid segment located in the catalytic domain of PI3Kδ. In subsequent functional studies involving ectopic overexpression of the short variant (see below), we concentrated our efforts on the variant missing exon 20 given the possibility that kinase activity may be affected.

### *PIK3CD-S* isoform augments invasion and proliferation

**Figure 4:**
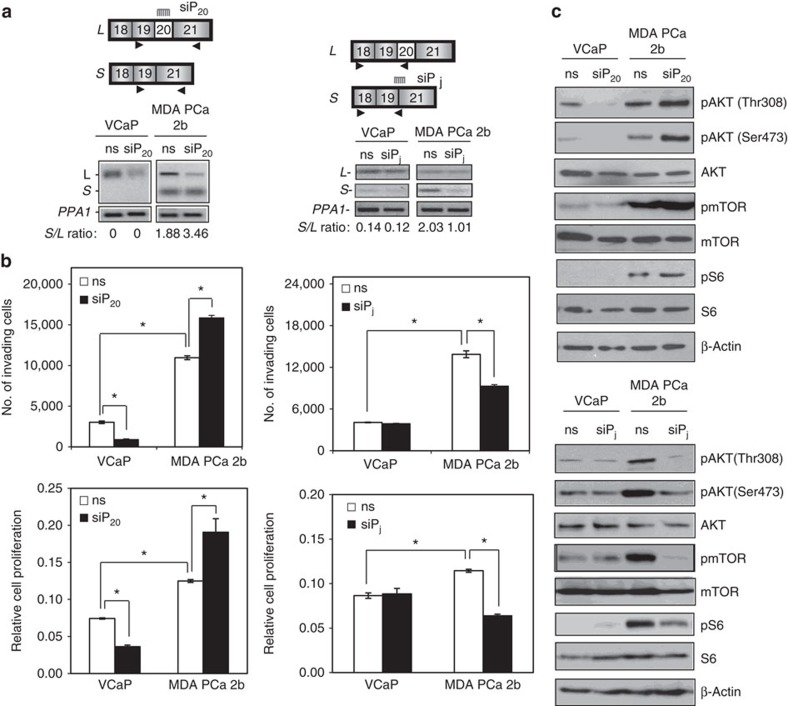
Selective knockdown of the AA-enriched *PIK3CD-S* variant or race-independent *PIK3CD-L* variant has opposing effects on tumorigenesis and AKT/mTOR signalling in AA PCa cells. (**a**) RT-PCR of *PIK3CD-L* and *PIK3CD-S* variants following knockdown in the EA PCa cell line VCaP and AA PCa cell line MDA PCa 2b. Specific knockdown of the *PIK3CD-L* variant (left panel) was accomplished with an exon 20-targeting siRNA (siP_20_), while knockdown of the *PIK3CD-S* variant (right panel) was achieved with an siRNA targeting the region spanning exons 19 and 21 (siP_j_). Closed arrowheads below exons represent primer location for qRT-PCR validation of alternatively spliced transcript knockdowns. Knockdown efficiency of siP_20_ and siP_j_ siRNAs was determined by the *S/L* ratio derived from RT-PCR reactions; ns, nonsense siRNA treatment. Representative images of *n*=4 independent knockdown experiments. (**b**) Proliferation and invasion of VCaP and MDA PCa 2b following knockdown of the *PIK3CD-S* (left panel) or *PIK3CD-L* variant (right panel). Data presented as mean±s.e.m. from at least three independent experiments for each treatment group. *P<0.05 by ANOVA with *post hoc* Tukey. Variance was similar among groups being compared. (**c**) Western blot analysis of AKT/mTOR signalling following knockdown of the *PIK3CD-L* (top panel) or *PIK3CD-S* variant (bottom panel) in VCaP and MDA PCa 2b. Level of AKT, mTOR and ribosomal S6 kinase activities is reflected by the amount of phospho-AKT (pAKT), phospho-mTOR (pmTOR) and phospho-ribosomal protein S6 (pS6) immunoblotting, respectively. β-Actin served as loading control. Representative images from at least three independent western blot experiments.

We hypothesized that the splice variants specific or enriched in AA PCa may contribute to a more aggressive oncogenic phenotype. To test this, we designed exon-specific and exon junction-specific short interfering RNAs (siRNAs) to target *PIK3CD-L* and *PIK3CD-S*, respectively, in EA and AA PCa cell lines and examined the functional consequences of these knockdowns on cell proliferation and invasion. A similar strategy was applied to investigate the biological significance of the variants of *FGFR3*, *TSC2* and *RASGRP2*. VCaP and MDA PCa 2b cells were used as population-specific PCa models, as these two cell lines represent bone metastases derived from castration-resistant EA and AA PCa patients, respectively^35^,^36^. Transfection of VCaP cells with exon 20-specific siRNA (siP_20_) successfully knocked down *PIK3CD-L* expression by >8-fold compared with nonsense siRNA (Fig. 4a, left panel), resulting in a significant loss of proliferative and invasive function in VCaP cells (Fig. 4b, left). Conversely, in MDA PCa 2b cells, a >5-fold knockdown of *PIK3CD-L* increased the ratio of *PIK3CD-S/PIK3CD-L* expression by nearly twofold (Fig. 4a, right panel; 1.88 *S/L* ratio for nonsense versus 3.46 *S/L* ratio for siP_20_-transfected cells), and this ‘enrichment’ of AA-enriched *PIK3CD-S* subsequently enhanced proliferation and invasion of the AA cell line (Fig. 4a, right panel). Moreover, MDA PCa 2b cells exhibited significantly higher basal invasive and proliferative capacities compared with VCaP cells (proliferation and invasion of siNS-transfected MDA PCa 2b versus siNS-transfected VCaP; Fig. 4b, left and right panels). To further evaluate the functional impact of *PIK3CD-S* expression on cell proliferation and invasion, the EA and AA PCa cell lines were transfected with siP_j_ (siRNA specifically targeting the junction of exons 19 and 21). Transfection of siP_j_ had no effect on VCaP proliferation and invasion, as expected since this EA line does not significantly express *PIK3CD-S* (Fig. 4a,b; right panels). On transfection of MDA PCa 2b cells with siP_j_, *PIK3CD-S* expression was significantly knocked down (Fig. 4a, right), resulting in a loss of cell proliferation and invasion (Fig. 4b, right). Taken together, these results suggest that *PIK3CD-S* is the more aggressive variant, promoting PCa proliferation and invasion to a greater extent than *PIK3CD-L*.

Several additional exon-specific siRNAs were designed to test whether other AA-specific/-enriched splice variants also functionally contribute to greater PCa aggressiveness. SiRNAs targeting exon 14 (*siFGFR3-ex14*), exon 20 (*siTSC2-ex20*) and exon 11 (*siRASGRP2-ex11*) were used to selectively suppress expression of *FGFR3-L*, *TSC2-L* and *RASGRP2-a* variants (predominately expressed in EA), respectively. Upon siRNA-mediated knockdown of *FGFR3-L*, *TSC2-L* or *RASGRP2-a* (Supplementary Fig. 6a–c, top panels) in MDA PCa 2b cells, the expression ratios of *FGFR3-S/FGFR-L*, *TSC2-S/TSC2-L* and *RASGRP2-b/RASGRP2-a* increased and correlated with augmented invasive and/or proliferative capacity of the AA-derived MDA PCa 2b cells (Supplementary Fig. 6a–c, bottom panels). Collectively, our *in vitro* studies strongly suggest that the AA-enriched splice variants *PIK3CD-S*, *FGFR3-S*, *TSC2-S* and *RASGRP2-b* promote PCa aggressiveness.

### *PIK3CD-S* isoform promotes activation of AKT/mTOR signalling

As PI3K plays a central role in the PI3K/AKT/mammalian target of rapamycin (mTOR) signalling pathway, we examined the ability of different PI3Kδ isoforms (encoded by *PIK3CD-L* and *PIK3CD-S*) to activate downstream signalling components within this pathway. SiRNA (siP_20_)-mediated knockdown of *PIK3K-L* expression (confirmed by qRT-PCR) led to a drastic decrease in AKT phosphorylation at Thr308 and Ser473 while moderately decreasing (approximately twofold) phosphorylation of mTOR in EA VCaP cells (Fig. 4c, top panel). In contrast, knockdown of *PIK3CD-L* in AA MDA PCa 2b cells (resulting in an increased *PIK3CD-S/-L* ratio confirmed by qRT-PCR) led to a sizable increase (two- to threefold) in phosphorylation of AKT, mTOR and ribosomal protein S6 (S6) (Fig. 4c, top panel).

In parallel experiments, siRNA (siP_j_)-mediated knockdown of *PIK3K-S* (confirmed by qRT-PCR) in MDA PCa 2b cells resulted in a drastic decrease in the phosphorylation status of AKT, mTOR and S6 (Fig. 4c, bottom panel). As expected, treatment of VCaP cells with the siRNA siP_j_ had negligible effects on AKT, mTOR and S6 phosphorylation, as this EA line does not significantly express *PIK3CD-S*. Taken together, the distinct phosphorylation patterns of AKT, mTOR and S6 in AA and EA PCa cell lines upon selective knockdown of either *PIK3CD-L* or *PIK3CD-S* again suggested that *PIK3CD-S* is the more aggressive variant, promoting oncogenic signalling.

### PI3Kδ-S isoform is resistant to SMIs

**Figure 5:**
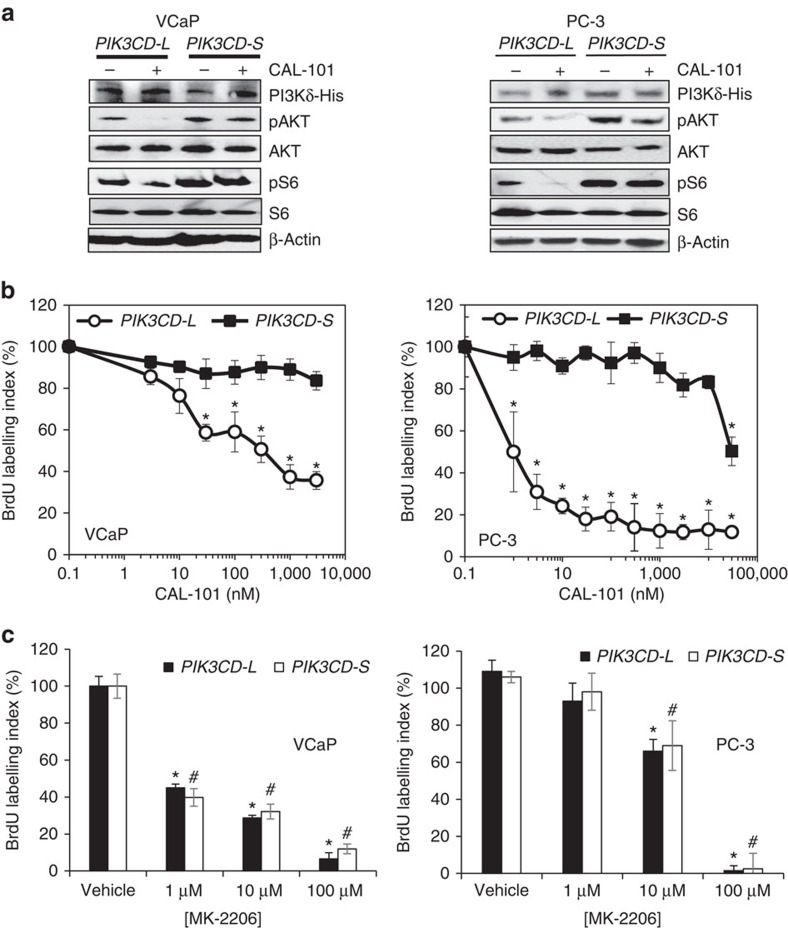
PI3Kδ-S but not PI3Kδ-L is resistant to small-molecule inhibition of PI3K/AKT/mTOR signalling and proliferation. (**a**) Assessment of PI3K/AKT/mTOR signalling following treatment with vehicle (saline) or CAL-101 (100 nM, 24 h). PI3K/AKT/mTOR signalling was assessed by western blot analysis with phospho-antibodies to AKT (pAKT) and S6 ribosomal protein (pS6). β-Actin served as a loading control. His-tag antibody was used to demonstrate equal expression of His-tagged variant PI3Kδ protein in stably transfected cell lines. Representative images from at least three independent western blot experiments. Unprocessed western images shown in Supplementary Fig. 12. (**b**) Proliferation in VCaP and PC-3 cells stably overexpressing the *PIK3CD-S* variant or *PIK3CD-L* variant following treatment with vehicle (saline) or selective PI3Kδ small molecule inhibitor CAL-101 (24 h). Data presented as mean±s.e.m. from at least four independent experiments for each treatment group. *Significantly different from -S variant, *P*<0.05 by ANOVA with Dunnett’s *post hoc* test. (**c**) Treatment of *PIK3CD* variant-overexpressing cells with vehicle (saline) or selective AKT small molecule inhibitor MK-2206 (24 h). Proliferation was assessed using a 5-bromodeoxyuridine (BrdU) labelling assay. Data presented as mean±s.e.m. from at least four independent experiments for each treatment group. * Or ^#^significantly different from corresponding vehicle control, *P*<0.05 by ANOVA with Dunnett’s *post hoc* test. Variance was similar among groups being compared.

We tested whether pharmacological inhibition of PI3Kδ isoforms represented a potential strategy for ameliorating PCa aggressiveness. CAL-101, an SMI specific for PI3Kδ (refs 37, 38, 39), was employed to assess its inhibitory effects on oncogenic signalling and proliferation in EA VCaP and PC-3 cells that stably overexpressed the His-tagged PI3Kδ-S (excluding exon 20) or PI3Kδ-L isoform (including exon 20). Equivalent levels of PI3Kδ isoform expression in each cell line was confirmed by western blot with a His tag antibody (Fig. 5a). In both EA cell lines, ectopic overexpression of PI3Kδ-S was associated with a two- to threefold greater phosphorylation of AKT and S6 compared with ectopic overexpression of PI3Kδ-L (Fig. 5a, absence of CAL-101 treatment). CAL-101 (50 mg kg^−1^) induced a significant reduction in basal AKT and S6 phosphorylation (Fig. 5a) and a dose-dependent inhibition of proliferation in both EA cell lines overexpressing the PI3Kδ-L variant (Fig. 5b). In contrast, CAL-101 (50 mg kg^−1^) had negligible effects on inhibiting basal AKT signalling in EA PCa cell lines overexpressing PI3Kδ-S, as phosphorylation states of AKT and S6 were comparable to vehicle-treated cells (Fig. 5a). In agreement, 5-bromodeoxyuridine labelling assays demonstrated that proliferation of VCaP and PC-3 cells ectopically overexpressing PI3Kδ-S was greater than cells overexpressing PI3Kδ-L (Supplementary Fig. 7). Moreover, PI3Kδ-S-overexpressing VCaP and PC-3 cells were not effectively inhibited by CAL-101 treatment; only at extreme doses of CAL-101 (≥30 μM) was proliferative activity in PC-3 cells significantly impaired (Fig. 5b). In contrast, the AKT inhibitor MK-2206 (ref. 40) dose-dependently decreased proliferation in both PI3Kδ-S- and PI3Kδ-L-overexpressing VCaP and PC-3 cells (Fig. 5c). These results suggest that PI3Kδ-S-stimulated proliferation is resistant to CAL-101 inhibition in sharp contrast to PI3Kδ-L; while inhibition of AKT, which is downstream of PI3Kδ-S, effectively blocked proliferation.

**Figure 6:**
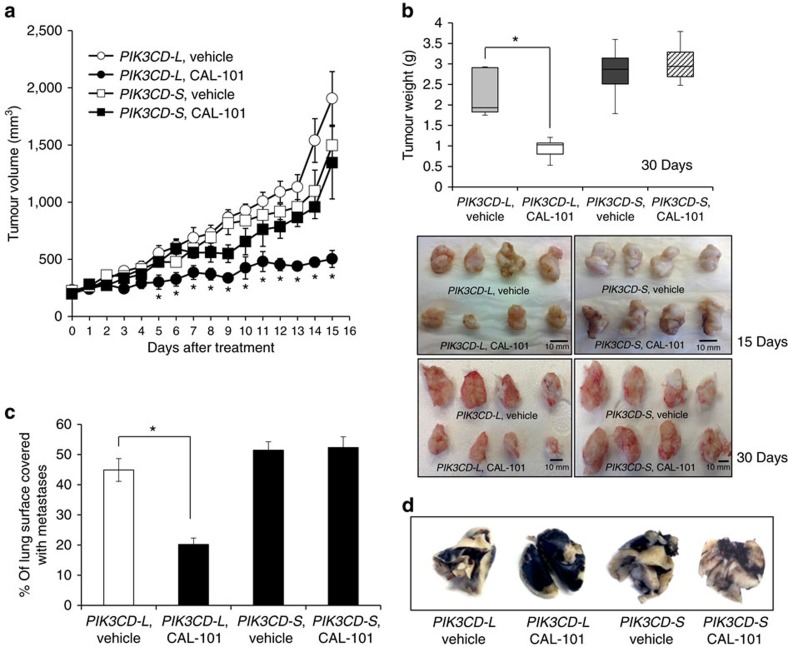
PI3Kδ-S but not PI3Kδ-L is resistant to small-molecule inhibition of xenograft growth and metastasis. (**a**) Growth of PC-3 cells stably overexpressing the *PIK3CD-S* variant in NOD-SCID mice is resistant to CAL-101 treatment (50 mg kg^−1^ i.p. 3 times a week). In contrast, growth of PC-3 cells stably overexpressing the *PIK3CD-L* variant is sensitive to CAL-101 treatment. Data represent the mean tumour size±s.e.m. of *n*=10 independent mice for each treatment group at each time point. *Significantly different from saline-treated group, *P*<0.05 by ANOVA with *post hoc* Tukey. Variance was similar among groups being compared. (**b**) Tumour weights and gross morphology of tumour xenografts from **a**. Box-and-whisker plot represents mean xenograft weight in mice after 30-day vehicle or CAL-101 treatment. *P<0.05 by ANOVA with Dunnett’s *post hoc* test; *n*=10 independent mice for each treatment group. Variance was similar among groups being compared. (**c**) Quantification of lung metastases in NOD-SCID mice. PC-3 cells stably overexpressing *PIK3CD-L* or *PIK3CD-S* were injected into the tail vein of NOD-SCID mice treated with vehicle or CAL-101 (50 mg kg^−1^ i.p. 3 times a week). After 8 weeks, lungs were collected and stained with India ink and bleached with Fekete’s solution for visualization of metastatic nodules (white-coloured areas). Data presented as mean±s.e.m. of *n*=10 for each treatment group. *P<0.05 by ANOVA with Dunnett’s *post hoc* test. Variance was similar among groups being compared. (**d**) Representative India ink-stained lungs from treatment groups analysed in **c**.

To examine the effects of *PIK3CD* splice variants on tumour growth *in vivo*, we subcutaneously injected 2 × 10^6^ PC-3 cells stably overexpressing equivalent amounts of the PI3Kδ-L or PI3Kδ-S (missing exon 20) isoform into the left hind flank of nonobese diabetic–severe combined immunodeficient (NOD-SCID) mice. Mice harbouring PI3Kδ-L*-*overexpressing or PI3Kδ-S-overexpressing PC-3 cell xenografts were administrated either vehicle (phosphate-buffered saline) or CAL-101 (50 mg kg^−1^) by daily intraperitoneal (i.p.) injection. CAL-101 treatment for 30 days significantly reduced the growth of PI3Kδ-L-expressing xenografts compared with the vehicle treatment (Fig. 6a,b). In contrast, mice with xenografts of PI3Kδ-S-expressing cells had negligible suppression of their xenograft growth following CAL-101 treatment compared with vehicle-treated animals (Fig. 6a,b).

We further examined the inhibitory effects of CAL-101 on PI3Kδ isoforms in an *in vivo* tumour metastasis model. The 1 × 10^6^ *PIK3CD-L-* or *PIK3CD-S-*overexpressing PC-3 cells were injected into the tail vein of NOD-SCID mice, and animals were subsequently administrated with vehicle or CAL-101 (50 mg kg^−1^) via i.p. injection (3 times per week). After 8 weeks, vehicle-treated mice carrying PI3Kδ-L-overexpressing cells developed prominent tumour metastases in the lungs (Fig. 6c,d), while the CAL-101 treatment group exhibited a >50% reduction (*P*<0.05) of metastases (Fig. 6c,d). In comparison, CAL-101 treatment failed to significantly inhibit tumour metastases in mice harbouring PI3Kδ-S-overexpressing cells (Fig. 6c,d). Noteworthy, the size of lung metastases (average area of nodules) in mice harbouring PI3Kδ-S-overexpressing cells was slightly greater (∼15%) compared with animals with PI3Kδ-L-overexpressing cells, although not statistically significant (*P*>0.05). Taken together, the *in vitro* and *in vivo* functional studies suggest that SMIs such as CAL-101 (competitive ATP binding inhibitors^39^,^41^) may be ineffective against the PI3Kδ-S isoform in AA PCa.

### Cell-free PI3Kδ isoform kinase assay

**Figure 7:**
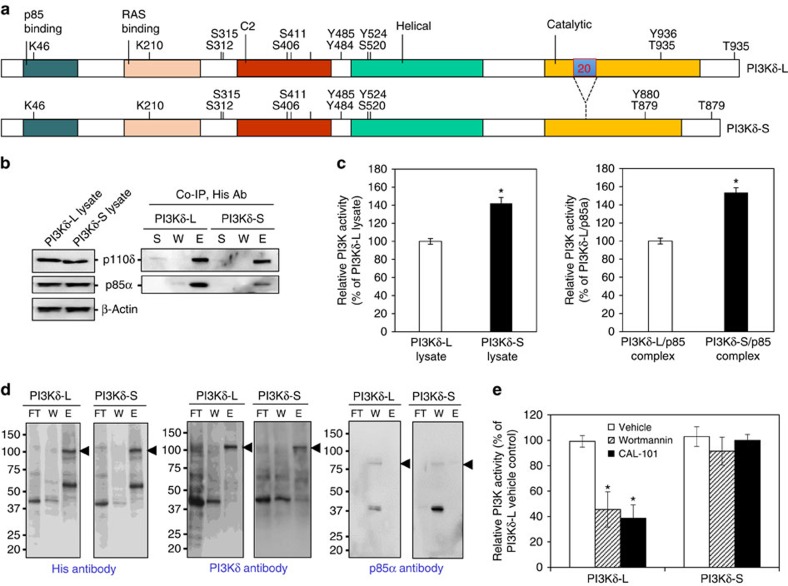
Cell-free kinase assay of PI3Kδ isoforms and small-molecule inhibition. (**a**) Schematic representation of protein domains of PI3Kδ-L and -S isoforms. Adaptor (p85)-binding, RAS binding, C2, helix and catalytic domains are highlighted. Phosphorylation and ubiquitination sites (S, T, Y and K) and the region encoded by exon 20 (56 amino acids) residing in the catalytic domain are indicated. (**b**) Co-immunoprecipitation (Co-IP) of His-tagged PI3Kδ/p85α complex from transfected PC-3 cells followed by western blot, and (**c**) PI3K activity assays. S, supernatant; W, wash fraction; E, eluted fraction. Anti-His antibody was used in the Co-IP experiments, and anti-His, anti-p85α and anti-actin antibodies were used in the western blotting. *Significantly different kinase activities in total lysates of PI3Kδ-S versus PI3Kδ-L-expressing cells, or purified PI3Kδ-S/p85α versus PI3Kδ-L/p85α complexes. *P*<0.05 using two-sided Student’s *t*-test. Data presented as mean±s.e.m. of *n*=4 for each treatment group. (**d**) Purification of His-tagged PI3Kδ-L and -S isoforms. Western blot analysis of Ni-NTA resin-purified PI3Kδ isoforms from transfected PC-3 cells using His and PI3Kδ antibodies. FT, flow-through; W, wash fraction; E, eluted fraction. Closed arrowheads indicate PI3Kδ isoforms. (**e**) Cell-free kinase assay of L and S isoforms of PI3Kδ in the presence of vehicle (phosphate-buffered saline (PBS)), 100 nM wortmannin or 100 nM CAL-101. *Significantly different from vehicle control-treated PI3Kδ-L isoform. *P*<0.05 by ANOVA with Dunnett’s *post hoc* test. Data presented as mean±s.e.m. from at least four independent experiments for each treatment group. Blots in **b**,**d** are representative from at least three independent experiments with similar results. Variance was similar among groups being compared. Unprocessed western images are shown in Supplementary Fig. 13.

The consequence of excluding exon 20 (168 bp) in the *PIK3CD-S* variant is an in-frame deletion of 56 amino acids (residues 810–865) in the catalytic domain of the PI3Kδ-S isoform (Fig. 7a). To gain further insight into the functional differences between PI3Kδ isoforms, the interaction of PI3Kδ-L and -S with regulatory subunit p85α was investigated. Whole cell lysates from transfected PC-3 cells overexpressing p85α and either His-tagged PI3Kδ-S or PI3Kδ-L were subjected to western analysis, demonstrating that each cell line expressed equivalent levels of their respective PI3Kδ isoform as well as equal p85α expression (Fig. 7b, left panel). Interestingly, co-immunoprecipitation (co-IP) of the PI3Kδ/p85α complex from whole cell lysates using an anti-His antibody demonstrated that p85α bound with three- to fourfold greater proficiency to PI3Kδ-L compared with p85α binding to PI3Kδ-S (Fig. 7b, right panel, column E). Binding proficiency was inversely correlated with PI3Kδ isoform kinase activity (Fig. 7c, right panel).

Next, PI3Kδ isoforms were purified from the lysates of PC-3 cells overexpressing either His-tagged PI3Kδ-S or PI3Kδ-L using Ni-NTA resin columns. As shown in Fig. 7d (left and middle panels), PI3Kδ-S and -L purification was verified by western blotting using anti-His or anti-PI3Kδ antibody. Moreover, the Ni-NTA resin column approach resulted in the isolation of PI3Kδ isoforms that were no longer bound to p85α (Fig. 7d; far right panel, column E). Early reports have demonstrated that PI3Kα/p85 and PI3Kβ/p85 complexes are obligate and extremely stable, being able to withstand high concentrations of urea or detergent^42^,^43^,^44^. Our finding that PI3Kδ and p85α co-exist as monomers and complexes was unexpected. It should be noted, however, that the nature of interaction between PI3Kδ and p85 is less well established, as these two proteins have been shown in separate studies to either form a stable obligate complex^42^ or coexist complexed together and uncomplexed from each other^45^.

Purified PI3Kδ isoforms (minus p85α) were incubated with vehicle, nonselective PI3K inhibitor wortmannin (100 nM)^46^ or PI3Kδ-specific inhibitor CAL-101 (100 nM), and subjected to a PI3K activity assay. In the absence of bound p85α, kinase activity of PI3Kδ-L was equivalent to PI3Kδ-S (Fig. 7e, compare vehicle treatments). In agreement, siRNA-mediated knockdown of p85α in wild-type VCaP and PC-3 cells was associated with an increase in invasive activity (Supplementary Fig. 8). Remarkably, wortmannin and CAL-101 significantly inhibited the activity of the PI3Kδ-L isoform, but not the PI3Kδ-S isoform (Fig. 7e). These results demonstrate that PI3Kδ-S maintains kinase activity even in the presence of SMIs, supporting the *in vitro* and *in vivo* results (Figs 5 and 6).

## Discussion

The phenomenon of DS, much less global DS events, has not been adequately explored as a possible mechanism underlying PCa disparities. Potential involvement of the constitutively active *AR-V7* splice variant in PCa disparities has been suggested in a recent study. Selective downregulation of miR-212 observed in AA PCa is correlated with upregulation of splicing factor hnRNP-H1, upregulation of *AR-V7* and antiandrogen resistance in PCa cell lines^47^. In contrast to this localized splicing event, our study reveals that DS on a global scale may be a critical molecular mechanism underlying PCa disparities. In a comparison of AA PCa versus EA PCa, DS events were found to be highly prevalent in cancer-associated genes and pathways (Supplementary Data 1 and 2). Interestingly, the number of genes harbouring predicted DS events (2,520 genes) was ∼3 × greater than the number of differentially expressed, but not differentially spliced, genes (886 genes). These findings have two major implications. First, alternative/aberrant splicing of pre-mRNAs may have a greater role than differential gene expression in driving PCa disparities. Second, predicted DS events identified in our study were statistically overrepresented in oncogenic signalling pathways. In many cases, these same pathways are known to harbour a preponderance of gene mutations across different cancer types^27^,^28^,^29^. Hence, DS adds another layer of complexity to the existing molecular repertoire (gene mutation, expression, methylation^7^) driving AA PCa aggressiveness.

Studies on AS indicate that approximately half of such events occurring in the coding sequence are in-frame, while the remaining events are frameshifts leading to truncated or extended C-terminal proteins^48^,^49^. Remarkably, 70% of AA-enriched/-specific variants in our composite oncogenic signalling pathway (Fig. 2) were in-frame, including *PIK3CD-S*, *FGFR2-S, FGFR3-S*, *TSC2-S*, *RASGRP2-b*, *ATM-S* and *GSK3-S* (Supplementary Table 2). In comparison, only 27.3% of EA-enriched/-specific DS events in our composite oncogenic signalling pathway exhibited in-frame preservation (Supplementary Table 2), while the remaining EA-enriched DS variants, including *ITGA4-S*, *MET-S*, *NF1-S*, *RASGRP2-a*, *mTOR-S* and *BAK1-S*, were frame-shifted. Why the vast majority of DS events appear to be in-frame in AA PCa versus frame-shifted in EA PCa remains unresolved (Supplementary Table 1). Presumably, the preponderance of AA in-frame events detected in oncogenic signalling pathways may be contributing to the more aggressive nature of AA PCa. Possible mechanisms that could drive differences in AS events include differential expression of *trans*-acting splicing factors^47^ and/or single-nucleotide polymorphisms in *cis*-acting splicing elements of alternatively spliced genes^50^. In fact, a number of splicing factor mRNAs appear to be overexpressed (*SRSF2*, *SRSF7*) in AA PCa compared with EA PCa^9^,^10^. Regarding the in-frame variants (*PIK3CD-L*, *FGFR3-L* and *TSC2-L*) detected in EA PCa, each conferred a less aggressive oncogenic phenotype compared with the corresponding in-frame variants detected in AA PCa (*PIK3CD-S*, *FGFR3-S* and *TSC2-S*).

Approximately one-third of the AA-enriched/-specific variants identified in AA PCa were likewise present in patient-matched NP specimens, whereas the remaining AA-enriched/-specific variants found in PCa were absent in patient-matched NP specimens and thus appear to be *de novo* events (occurring as NP evolved into PCa). Accordingly, the AA-enriched/-specific variants already present in NP specimens have the potential to serve as inherent ‘at-risk alleles’ for poor PCa prognosis in AAs. In comparison, the *de novo* appearance of tumour-specific variants may drive poorer outcomes. *PIK3CD-S* would be an example of a potential AA ‘at-risk allele’ contributing to increased PCa aggressiveness upon disease presentation. Indeed, ectopic overexpression of the AA-enriched *PIK3CD-S* in PCa cell lines was demonstrated to enhance oncogenic potential (increased invasion, proliferation and AKT/mTOR signalling) compared with the corresponding EA-enriched *PIK3CD-L*. Moreover, genetic manipulation of AA MDA PCa 2b cells to favour expression of the *-S* variant over the *-L* variant likewise increased oncogenic behaviour. Conversely, genetic manipulation in the opposite direction decreased oncogenic behaviour. Interestingly, survival plots generated from The Cancer Genome Atlas (TCGA) RNA-sequencing data demonstrate that a high *S/L* ratio is associated with significantly worse survival for PCa and trending for worse survival in both breast and colon cancer (Supplementary Fig. 9). Survival plots were not stratified by race as this information is not currently available in TCGA. Given the number of patients analysed, it seems highly probable that high *S/L* ratio values may also be associated with a subset of EA patients, suggesting that *PIK3CD-S* may be useful in predicting survival in all patients irrespective of race. Besides *PIK3CD-S*, an additional 732 potential ‘at-risk alleles’ (for example, *ITGA4-L*, *MET-L*) were identified that may be associated with poor PCa prognosis in AAs. Further experimentation is needed to investigate whether these variants can serve as novel biomarkers to address PCa disparities. In contrast to the ‘at-risk alleles’, AA-enriched variants *FGFR-S* and *TSC2-S* were detected in AA PCa, but not in patient-matched NP specimens. The appearance of these *de novo* variants during PCa formation may contribute to driving the more aggressive PCa phenotype observed in the AA population, since *in vitro* genetic manipulation favoring expression of these *-S* variants over the -*L* variants promoted oncogenesis in MDA PCa 2b cells. It is noteworthy that three PCa-associated splice variants identified in previous studies, *Bcl-xL*, *FGFR2-IIIc* and *TMPRSS2-ERG+72* (refs 14, 15, 20), did not exhibit DS in our AA PCa versus EA PCa comparison (Supplementary Data 1), suggesting that these variants may contribute to PCa progression/aggressiveness in a race-independent manner.

The identification of *PIK3CD-S*, a variant newly discovered and cloned in our study, as an ‘at-risk allele’ for PCa aggressiveness is germane given that PI3K signalling is aberrantly activated in a variety of cancers and PI3K inhibitors have been developed as targeted therapeutics^51^,^52^. Class IA PI3Ks consist of three isoforms, including PI3Kα, PI3Kβ and PI3Kδ. Unlike ubiquitously expressed PI3Kα and PI3Kβ, PI3Kδ appears to be preferentially expressed in leukocytes^53^,^54^. Previous studies have revealed a crucial role of PI3Kδ in lymphoid and myeloid malignancies^39^,^55^. Interestingly, accumulating evidence suggests a functional role of PI3Kδ in promoting nonhaematologic tumours as well. For example, overexpression of *PIK3CD* mRNA and/or PI3Kδ protein has been detected in glioblastoma^32^, neuroblastoma^30^, breast cancer^33^ and PCa^31^, and *PIK3CD* overexpression has been implicated in promoting cell growth/survival in breast cancer and neuroblastoma^30^,^33^. Consistent with these findings, our immunohistochemistry experiments using a pan-PI3Kδ antibody likewise revealed strong expression of PI3Kδ protein in PCa specimens as well as PCa, breast cancer and colon cancer cell lines (Supplementary Fig. 10). Importantly, our study provides greater granularity by being the first to demonstrate the relationship between expression of a race-enriched *PIK3CD* splice variant and cancer aggressiveness as well as resistance to SMIs targeting PI3Kδ.

Aberrant pre-mRNA splicing has recently been demonstrated to mediate therapeutic resistance in multiple cancer types. For example, the constitutively active *AR-V7* variant (lacking exonic sequences encoding the ligand binding domain) confers resistance to enzalutamide and abiraterone acetate in castration-resistant PCa patients^56^. In addition, melanoma patients harbouring *BRAF* splice variants encoding protein isoforms that are missing the RAS-binding domain exhibited resistance to the RAF inhibitor vemurafenib^57^. Noteworthy, these studies did not investigate whether variant expression and therapeutic responsiveness stratified along racial lines. We now provide evidence that AA-enriched *PIK3CD-S* imparts PCa cell lines with significant resistance to SMIs targeting PI3Kδ, as demonstrated in both *in vitro* assays and preclinical mouse models of PCa. This short variant is missing exon 20, encoding a 56-amino acid segment that is present in *PIK3CD-L*. Amino acids residing in the exon 20-encoded cassette appear critical for the docking of CAL-101 and wortmannin. Indeed, molecular modelling studies predict that Glu826 and Val828 (missing in PI3Kδ-S) undergo hydrogen bonding with CAL-101 (ref. 58). Noteworthy, overall response of indolent lymphoma and chronic lymphocytic leukaemia to CAL-101 ranges from 48 to 81% (refs 59, 60, 61). Given our findings, it would be of interest to determine whether patients with primary resistance harbour malignant cells expressing CAL-101-resistant PI3Kδ-S, while responsive patients harbour malignant cells expressing CAL-101-sensitive PI3Kδ-L.

P85 regulatory subunits are known binding partners of class I PI3Ks, resulting in protein stabilization and suppression of basal kinase activity^62^,^63^. Somatic mutations in *PIK3R1* (encoding p85α) have been identified that abrogate the inhibitory action of p85α on PI3Kα in cancers^64^,^65^. Our cell-free assays demonstrated that p85α binds more efficiently with PI3Kδ-L compared with PI3Kδ-S. This interaction appears to be responsible for the lower kinase activity exhibited by PI3Kδ-L, as disruption of binding led to a long isoform with increased kinase activity comparable to PI3Kδ-S (Fig. 7c,e). The amino acid Asn334 located on the N-terminal side of PI3Kδ has been postulated to serve as a critical contact point with p85α (ref. 66). Our findings suggest that amino acids 810–865, encoded by exon 20 and missing in PI3Kδ-S, may also contain essential amino acids required for efficient coupling to p85α. Alternatively, amino acids 810–865 permits PI3Kδ-L to adopt a conformation where Asn334 (and other amino acids) is available to interact with p85α.

The identification and functional validation of global AS in cancer pathogenesis remains challenging and largely unexplored. We have undertaken such an analysis in the context of race-related aggressive PCa and identified a large number of DS events in cancer-associated pathways in EA and AA PCa, with a subset of these events also being detected in patient-matched NP specimens. These events will have both biological and clinical consequences, case in point *PIK3CD-S*. The identification of novel splice variants as biomarkers and/or development of therapeutics targeting protein isoforms have the potential to reduce cancer disparities.

## Methods

### Materials

EA PCa cell lines LNCaP (CRL-1740), VCaP (CRL-2876) and PC-3 (CRL-1435), and AA PCa cell line MDA PCa 2b (CRL-2422) were obtained from the American Type Tissue Collection (ATCC, Manassas, VA, USA), authenticated at the ATCC by short tandem repeat profiling of multiple unique genetic loci, and tested negative for mycoplasma. Primer sequences for RT-PCR are provided in Supplementary Table 3. The siRNAs were purchased from GE Dharmacon (Lafayette, CO, USA) and sequences are as follows: nonsense, 5′-CCA AAUUAUACCUACAUUGCU-3′; siP_20_, 5′-CCAACAUCCAACUCAACAA-3′; siP_j_, 5′UGAGGGAGGCCCUGGAUCGA-3′; siF, 5′-CUCGACUACUACAAGAAGA-3′; siTSC2-ex20, CUGCGCUAUAAAGUGCUCA-3′; siRASGRP2-ex11, 5′-CCACAUCUCACAGGAAGAA-3′. siPIK3R1 Smart Pool (5′-AGUAAAGCAUUGUGUCAUA-3′, 5′-CCAACAACGGUAUGAAUAA-3′, 5′-GACGAGAGACCAAUACUUG-3′, 5′-UAUUGAAGCUGUAGGGAA A-3′).

### Collection of PCa clinical specimens

Prostate biopsy samples were collected at the George Washington University Medical Faculty Associates according to an institutional review board-approved protocol (IRB no. 020867). Informed consent was obtained from all study participants. High-quality PCa and patient-matched normal prostate (NP) biopsy cores from each of 20 AA and 15 EA primary PCa patients were collected and processed for the exon array analysis. PCa cores were determined by a pathologist to have Gleason scores of 6–7 (17 AA and 13 EA) or 8–9 (3 AA and 2 EA), while NP cores were diagnosed negative for cancer. There were no significant differences (*t*-test, *P*>0.05) between the two racial groups with respect to age (average age for AAs was 62.3±8.2, average age for EAs was 63.3±9.2) and Gleason score (range 6–8; Fisher’s exact test, *P*>0.05). No distant metastasis was detected in the enrolled patients.

### Exon array and statistical analyses

Total RNA was purified from PCa and patient-matched NP biopsy cores using the RNeasy micro kit as per manufacturer’s protocol (Qiagen, Valencia, CA, USA). Briefly, total RNA samples were extracted using Trizol reagent, then treated with DNase I and further purified by RNeasy MinElute spin column. High-quality RNA isolation was confirmed by using the Agilent Bioanalyzer as per the manufacturer’s protocol (Agilent Technologies, Santa Clara, CA, USA). For exon array analysis, 1 μg of purified RNA sample from each biopsy core was interrogated with the Affymetrix Human Exon 1.0 ST GeneChip (Santa Clara, CA, USA). Exon microarray data can be assessed at GEO (Gene Expression Omnibus) using accession number GSE64331. The exon array raw data were subjected to quantile normalization, GC-content adjustment, RMA background correction and log_2_ transformation using Partek Genomics Suite 6.6 software (Partek Incorporated, St Louis, MO, USA). Detection of differential expression at the gene level (gene-wise analysis) was performed in Partek using the One-Step Tukey’s Biweight algorithm for detection of outlier probe-sets. Statistical analysis of exon expression data was based on ANOVA with multiple-correction testing using 10% false discovery rate (FDR)^67^ criterion. DS events were modelled using the AS ANOVA algorithm^26^ implemented in Partek together with selection of probe-sets exhibiting significant AS score determined at a 2% FDR. Principal component analysis plots and two-dimensional hierarchical clustering of exon-level data were performed using Partek. DS events were tested for statistical overrepresentation in canonical signalling pathways by Fisher’s exact test using the Ingenuity Pathway Analysis (IPA) program (Ingenuity Systems, Redwood City, CA, USA).

### RT-PCR validation of AS variants in AA and EA PCa

QRT-PCR was performed using the 7300 Real-Time PCR System (Applied Biosystems, Foster City, CA, USA) to validate and quantify AS events. Primers were designed to amplify the flanking regions of skipped exons or the junctions across catenated exons of variant mRNAs (Fig. 3b). Amplified RT-PCR products were quantified and normalized to housekeeping genes, *EIF1AX* and *PPA1*, using the ΔΔCt approach^9^,^10^. Primer sequences for RT-PCR validation are listed in Supplementary Table 3.

### Molecular cloning of *PIK3CD-S* and *PIK3CD-L* variants

RT-PCR was performed to amplify *PIK3CD-L* and *PIK3CD-S* transcript variants from purified RNA of PC-3, VCaP and MDA PCa 2b cells (ATCC, Manassas, VA). PC-3 and VCaP cells were maintained in Dulbecco’s modified Eagle’s medium (DMEM; Life Technologies, Gaithersburg, MD, USA) supplemented with 10% fetal bovine serum (FBS), while MDA PCa 2b cells were grown in BRFF-HPC-1 medium (AthenES, Baltimore, MD, USA) supplemented with 20% FBS. All the cell lines were grown at 37 °C and 5% CO_2_. Primers were designed according to the National Center for Biotechnology Information (NCBI) reference sequences of *PIK3CD* mRNA (NM_005026.3). The forward primer contained the start codon (bold) (5′-**ATG**CCCCCTGGGGTGGACT-3′) and the reverse primer was upstream of the stop codon (5′-CTGCCTGTTGTCTTTGGACA-3′). Full-length PCR products were ligated into pcDNA3.1/V5-His TOPO vector (K4800-01, Invitrogen, Grand Island, NY, USA) using the manufacturer’s protocol. A total of 8–10 independent clones were selected for each of the amplified *PIK3CD-L* and *PIK3CD-S* variants and sequence verified. The consensus sequences of *PIK3CD-S* and *PIK3CD-L* mRNAs were deposited to GeneBank (accession number KU612116 and KU612117). The plasmids pcDNA3.1-PIK3CD-L/V5-His and pcDNA3.1-PIK3CD-S/V5-His were individually transfected into the PCa cell lines (VCaP and PC-3) using the cationic lipid-mediated method^9^ to establish stable cell lines overexpressing *PIK3CD-L* or *PIK3CD-S.*

### SiRNA-mediated knockdown in PCa cell lines

VCaP and MDA PCa 2b cells were grown in DMEM with 10% FBS for 24 h, and then were transfected for 24 h with siRNAs (50 nM) designed to target splice variants of *PIK3CD*, *FGFR3*, *TSC2* or *RASGRP2* using DharmaFECT4 transfection reagent (Dharmacon), according to the manufacturer’s protocol. The *in vitro* functional assays, including cell proliferation and invasion, were performed following siRNA transfections for 24 h. Cell proliferation and invasion assays were performed using 5-bromodeoxyuridine Cell Proliferation Assay kit (Calbiochem, Billerica, MA, USA) and the Matrigel Invasion Chambers (BD Biosciences, San Jose, CA, USA), respectively, as per the manufacturers’ protocol^9^,^10^.

### Antibodies

Antibodies used in western blot analysis were rabbit monoclonal antibodies for pAKT^Tyr308^, pAKT^Ser473^, AKT, pmTOR, mTOR, pS6 and S6 (2965, 4058, 4691, 2971, 2983, 4857 and 2983, Cell Signaling Technology, Danvers, MA, USA), rabbit polyclonal antibodies for His-tag (ab18184, Abcam, Cambridge, MA, USA), HA-tag, PI3Kδ, p85α and β-actin (sc-7392, sc-55589, sc-1637 and sc-4778, Santa Cruz Biotechnology, Santa Cruz, CA, USA). Horseradish peroxidase-conjugated secondary antibodies for rabbit and mouse IgG were purchased from Southern Biotech (Birmingham, AL, USA).

### *In vivo* xenograft and metastasis models

All animal work was approved by the George Washington University institutional animal care and use committee (protocol A272). Male NOD-SCID mice, 4–6 weeks old, were purchased from the Jackson Laboratory (Bar Harbor, ME, USA). To establish a PCa xenograft model, 2 × 10^6^ PC-3 cells stably overexpressing *PIK3CD-L* or *PIK3CD-S* were subcutaneously injected into the left flank of NOD-SCID mice. Tumour xenograft growth was measured with calipers and the volume was determined as 1/2 × length × width^2^. Mice were randomized into groups once the average tumour size reached ∼200 mm^3^ and treated with vehicle (phosphate-buffered saline) or CAL-101 (50 mg kg^−1^) through daily i.p. injections. After 30 days, mice were euthanized and the dissected xenografts were photographed and weighed using a blinded design.

To establish the PCa metastasis model, 1 × 10^6^ PC-3 cells stably overexpressing *PIK3CD-L* or *PIK3CD-S* were injected into the tail vein of NOD-SCID mice. The mice were then treated with vehicle or CAL-101 (50 mg kg^−1^) via i.p. injections, 3 times a week. After 8 weeks, lungs of mice were collected and stained with India ink and bleached with Fekete’s solution (70% ethanol, 3.7% formaldehyde, 0.75 M glacial acetic acid). India ink-stained lungs were photographed and lung metastases were quantified using the NIH ImageJ program^68^.

### Purification of His-tagged PI3Kδ protein

PC-3 cells stably overexpressing *PIK3CD-L* or *PIK3CD-S* were maintained in DMEM (Life Technologies) supplemented with 10% FBS. After growing the cells for 24 h, cell extracts were prepared and His-tagged PI3Kδ protein was purified using a column HisPur Ni-NTA purification kit (Pierce Biotechnology, Rockford, IL, USA). Briefly, cell lysates were mixed with Ni-NTA resin and incubated at room temperature for 30 min. After incubation, the resin was washed with wash buffer (25 mM imidazole, pH 7.4) and applied to a HisPur Ni-NTA spin column, centrifuged and wash buffer eluate discarded after centrifugations. His-tagged proteins were eluted from the resin by adding one-resin-bed volume of elution buffer (250 mM imidazole, pH 7.4). The purified PI3Kδ-His protein was mixed with 2 × Laemmli sample buffer, boiled and analysed by immunoblotting.

### Co-IP of PI3Kδ/p85 complex

Plasmids pcDNA3.1-PIK3CD-S/V5-His (or pcDNA3.1-PIK3CD-L/V5-His) and pSV-p85α (Addgene, Cambridge, MA, USA) were co-transfected into PC-3 cells. After growing the cells for 48 h, the co-transfected cells were collected and cells were lysed with RIPA lysis buffer (Santa Cruz Biotechnology). The cell lysates were then subjected to Co-IP assays with anti-His antibody (ab18184, Abcam) and immobilized on protein G-Sepharose beads (Thermo Scientific, Waltham, MA, USA). Cell lysates and precipitates were subjected to western blotting, and visualized by enhanced chemiluminescence system (Thermo Scientific, Waltham, MA, USA).

### *In vitro* assay of PI3Kδ activity

PI3Kδ activity was evaluated with a PI3K activity/inhibitor assay kit (Millipore, Billerica, MA, USA) according to the manufacturer’s instructions. Briefly, purified His-tagged PI3Kδ-L or PI3Kδ-S isoform was pretreated with the PI3Kδ inhibitor (100 nM of wortmannin or 100 nM of CAL-101) or vehicle in 96-well plates for 10 min and subjected to a competitive ELISA. PIP2 substrate and kinase reaction buffer were added to the pretreated His-tagged PI3Kδ-L or PI3Kδ-S isoform and incubated at room temperature for 1 h. After incubation, biotinylated PIP3 and GST-GRP1 working solutions were added to the wells and the reaction samples were further incubated at room temperature for 1 h. Plates were washed three times with 1 × Tris-buffered saline with Tween-20 (150 mM NaCl, 0.1% Tween-20, 50 mM Tris-Cl, pH 7.5) and incubated with streptavidin-horseradish peroxidase conjugate (1.25 mg ml^−1^) at room temperature for 1 h. After incubation, plates were washed three times and incubated with 100 μl of TMB (3,3′,5,5′-tetramethylbenzidine, 1 mg ml^−1^) substrate solution at room temperature for 5–20 min. Reactions were stopped by adding 100 μl of stop solution and plates read at 450 nm. The colourimetric signal was inversely proportional to the amount of PIP3 produced by PI3K activity and the relative amount of PIP3 produced was determined with a standard curve.

### Analysis of in-frame and out-of-frame exon skipping events

A total of 4,253 significant differentially expressed exons (2% FDR) were identified. Using a 1.5-fold cutoff, the number of differentially expressed exons (that is, exon skipping events) was narrowed down to 3,112 (corresponding to 2,520 DS genes in AA PCa versus EA PCa). The reference coordinates for the Affymetrix probe sets used to identify these skipped exons were cross-referenced with the Ensembl database, release GRCh37, resulting in final set of 2,517 well-curated, differentially expressed exons that corresponded to 1,484 DS genes (Supplementary Data 4). Based on the exon size and the modelled effect, the exon skipping events were classified as in-frame or frame-shift (Supplementary Data 4). A total of 1376 genes were included in this analysis. The distribution of in-frame and frameshift events was then compared between the EA and AA groups. We grouped the observations based on presence of at least one frameshift per gene. Notably, we observed significantly higher proportion of alternatively spliced genes without a frameshift in the AA group (34% in AA versus 27% in EA, *P*<0.005, Fisher’s exact test).

### Metrics for assessing reliability of global analysis of DS events

Affymetrix Exon GeneChip analysis has revealed ∼2,500 differential splicing events between AA and EA PCa. While we can only draw firm conclusions on a subset of differential splicing patterns that were validated by a second approach (that is, RT-PCR), the following metrics have been provided to allow evaluation of the overall reliability of Exon Genechip results:

1. *Validation success rate*. Eight of 9 genes (89%) identified by Exon GeneChip analysis to undergo differential RNA splicing between AA PCa and EA PCa were successfully validated by quantitative RT-PCR. The only gene that did not validate was *EPHA1*. In addition, 2 out of 2 genes (100%), defined not to exhibit differential RNA splicing (*GSK3A* and *ATM*), were successfully validated by quantitative RT-PCR. Aggregate success rate was 91% (10/11).
2. *P values.* The *P* values ranged from 1 × 10^−3^ to 1 × 10^−15^ for the 9 genes identified by Exon GeneChip analysis to undergo differential RNA splicing between AA and EA PCa specimens (*P*=6 × 10^−4^ for *ATM*). In comparison, *P* values for all genes identified by Exon GeneChip analysis to exhibit differential RNA splicing ranged from 3 × 10^−3^ to 1 × 10^−20^ (see Supplementary Data 1). Hence, *P* values of genes chosen for RT-PCR validation were representative of the entire range of *P* values associated with the complete set of ∼2,500 genes identified by Exon ChipGene analysis.
3. *Power calculations*. Based on the number of PCa patient samples interrogated by our Affymetrix Exon GeneChip arrays (*n*=15 to 20 patients per gene per race) and a computed s.d.=0.4, our findings correspond to >85% power to distinguish 1.5-fold changes at *P*<0.01.

### Estimation of *PIK3CD* isoform expression

Expression of the short and long isoforms of *PIK3CD* was determined using the method IsoformEx^69^. Briefly, isoform expression was estimated through the minimizing a weighted nonnegative least squares problem based on the exon expression. For the purpose of this analysis, the novel ‘short isoform’ was defined as any transcript missing exon 20 but having exons 19 and 21 concatenated; and the ‘long isoforms’ were defined as any transcripts with exons 19, 20 and 21 concatenated. Raw data for breast invasive carcinoma (BRCA), prostate adenocarcinoma (PRAD) and colorectal adenocarcinoma (COAD) were obtained from TCGA RNA-sequencing exon expression (https://tcga-data.nci.nih.gov/tcga/, accessed 22 January 2016). The ratio of short to long isoforms was calculated for survival analysis.

### Survival analysis

Disease-free survival data for BRCA, COAD and PRAD were obtained from TCGA clinical data (https://tcga-data.nci.nih.gov/tcga/, accessed 22 January 2016). Patients who did not have a relapse event during the study were considered as censored. The expression values of the short and long isoforms as well as the interaction term were used as predictors to fit the Cox proportional hazards regression model under L2-regularization, where the disease-free survival is the response variable. For each patient, a prognosis index score was computed from the Cox proportional hazards model^70^. Briefly, the patients were dichotomized into high- and low-risk groups according to the relapsed versus relapse-free ratio. The log-rank *P* value was then calculated to assess the statistically significant difference between the Kaplan–Meier curves of the high- versus low-risk groups.

### Data availability

The authors declare that all data supporting the findings of this study are available within the article and its Supplementary Information files or from the corresponding author on reasonable request. Sequences of *PIK3CD-S* and *PIK3CD-L* were deposited to GenBank (accession numbers KU612116 and KU612117).

## Additional information

**How to cite this article:** Wang, B.-D. *et al*. Alternative splicing promotes tumour aggressiveness and drug resistance in African American prostate cancer. *Nat. Commun.* **8**, 15921 doi: 10.1038/ncomms15921 (2017).

**Publisher’s note:** Springer Nature remains neutral with regard to jurisdictional claims in published maps and institutional affiliations.

## Supplementary Material

Supplementary Information

Supplementary Data 1

Supplementary Data 2

Supplementary Data 3

Supplementary Data 4
