# Corrigendum: Alternative splicing promotes tumour aggressiveness and drug resistance in African American prostate cancer

**DOI:** 10.1038/ncomms16161

**Published:** 2017-09-27

**Authors:** Bi-Dar Wang, Kristin Ceniccola, SuJin Hwang, Ramez Andrawis, Anelia Horvath, Jennifer A. Freedman, Jacqueline Olender, Stefan Knapp, Travers Ching, Lana Garmire, Vyomesh Patel, Mariano A. Garcia-Blanco, Steven R. Patierno, Norman H. Lee

Nature Communications 8: Article number: 15921 ; DOI: 10.1038/ncomms15921 (2017); Published: 06 30 2017; Updated: 09 27 2017

Contributions of the author Steven R. Patierno are not fully acknowledged in this Article. The Author Contributions section should have included the following:

‘S.R.P. was involved in the co-conception of the study, and was co-principal investigator of the initial portion of this study by obtaining NCI Center to Reduce Cancer Health Disparities grant supplement U01CA11637-05S2 (to S.R.P.) that supported initial experiments towards differential alternative splicing events, oncogenic splicing pathway, validation of differential alternative splicing and selective knockdown of PIK3CD L and S variants.’

