# Supplementary material for: Alternative splicing promotes tumour aggressiveness and drug resistance in African American prostate cancer
Source: Nat Commun. 2017 Jun 30;8:15921. doi: 10.1038/ncomms15921 (PMC5497057; doi:10.1038/ncomms15921)
Supplement: Supplementary Information [file ncomms15921-s1.pdf]

File name: Supplementary Information
Description: Supplementary Figures and Supplementary Tables

File Name: Supplementary Data 1
Description: Differentially spliced genes in 4-way analyses

File Name: Supplementary Data 2
Description: Categories of differentially spliced genes in diseases, molecular and cellular functions

File Name: Supplementary Data 3
Description: Significant pathways over-represented with DS in AA PCa vs EA PCa

File Name: Supplementary Data 4
Description: Exon list for differentially spliced genes in AA PCa and EA PCa

Supplementary Table 1. Splice variants encoding in-frame and out-of-frame protein products upon exon skipping in AA PCa and EA PCa.

Splice variant categories	Number of in-frame proteins after exon skipping		Number of out-of-frame proteins after exon skipping		Fisher's exact test *P*-value (two-tailed)
	AA PCa	EA PCa	AA PCa	EA PCa	
**All splice variants**	198	243	387	656	0.0053
**Splice variants of genes associated with cancer**	151	172	256	423	0.0073
**Splice variants of genes associated with all other diseases**	47	71	131	233	0.5103

Supplementary Table 2. Differential splicing of oncogenes and tumor suppressor genes in AA PCa vs. EA PCa, resulting in in-frame preservation or frame-shifting.

Gene symbol	exon/intron skipped	Length (bp)	In-frame or frame-shift	Exon skipping in AA PCa or EA PCa
**PIK3CD**	exon 8	90	in-frame	AA PCa
**PIK3CD**	exon 20	168	in-frame	AA PCa
**FGFR3**	exon 14	123	in-frame	AA PCa
**TSC2**	exon 20	123	in-frame	AA PCa
**ITGA4**	exon 18	151	frame-shift	EA PCa
**MET**	exon 11	219	frame-shift	EA PCa
**MET**	exon 12	147	frame-shift	EA PCa
**MET**	exon 13	157	frame-shift	EA PCa
**NF1**	exon 8	76	frame-shift	EA PCa
**BAK1**	exon 3	136	frame-shift	EA PCa
**RASGRP2**	exon 12	116	frame-shift	EA Pca
**RASGRP2**	exon 11	123	in-frame	AA PCa
**ATM**	exon 12	96	in-frame	AA PCa
**GSK3**	exon 8	96	in-frame	AA PCa
**EPHA1**	exon 13	62	frame-shift	AA PCa
**EPHA3**	intron 14	301	frame-shift	AA PCa
**FGFR2**	exon8	145	frame-shift	AA PCa
**mTOR**	intron 30	372	in-frame	EA PCa
**mTOR**	3' UTR	179	frame-shift	EA PCa
**TNFRSF11A**	5' UTR	93	in-frame	EA PCa
**TNFRSF21**	5' UTR	405	in-frame	EA PCa

Supplementary Table 3. Primer sequences for RT-PCR validation of splice variants in AA PCa and EA PCa.

Splice variants	Sequences of forward primer (5' to 3')	Sequences of reverse primer (5' to 3')
PIK3CD-L* and *-S	CTGAGCTCTCAGAAGACC	GCTCGCGGTTGATTCCAA
PIK3CD-L	GGTACTCCGTTCAGACACCA	GCTCGCGGTTGATTCCAA
PIK3CD-S	TGGACCTGAGGGAGGCCCT	GCTCGCGGTTGATTCCAA
FGFR3-L* and *-S	AAGGGTAACCTGCGGGAGTT	AGGTCGTGTGTGCAGTTGG
TSC2-L* and *-S	AGGTTGCGCAGTTAGCAGTT	GGTGCTTTCAGCAAAACCAT
ITGA4-L* and *-S	TTTTGTGCCCATGAAAATTG	TCAAGTTGTACCACGCCAGA
MET-L* and *-S	TACCACTCCTTCCCTGCAAC	TTTTCCAAGGACGGTTGAAG
NF-L* and *-S	AGGTTGCGCAGTTAGCAGTT	GGTGCTTTCAGCAAAACCAT
BAK1-L* and *-S	ATGGCTTCGGGGCAAGGC	TCATAGCGTCGGTTGATGTC
RASGRP2-a* and *-b	TGGAGCACATCGAGAAGATG	AGCGCAGGAAATAGGAAACC
	CAGATCATCCGTGGGAACTT	
RASGRP2-a	ATGGTTTCCTATTTCCTGCG	TGTGACTCCTAGGGGCTGAGT
RASGRP2-b	ATGGTTTCCTATTTCCTGCG	TTCTGGTCGAGGTCCCCAAA
ATM-L* and *-S	GCTACAGATTGCAACCCAATT	ACAGTTGCTCAAGCAACGTGT
GSK3A	ACAGGAGTATGCCAGTGTCCA	GGTGTAATCAGTGGCTCCAAA
EPHA1	AGGGCATAGCATCTGGCATGA	AGAATCAGAAGCGTGGAGTGC
EIF1AX	GTACTGGAGAGGGGAGAGCA	TGAAGCTGAGACAAGCAGGA
PPA1	GGCTGTTGTGGTGACAATGA	TGACTTTCCAGTCGGTTTCC

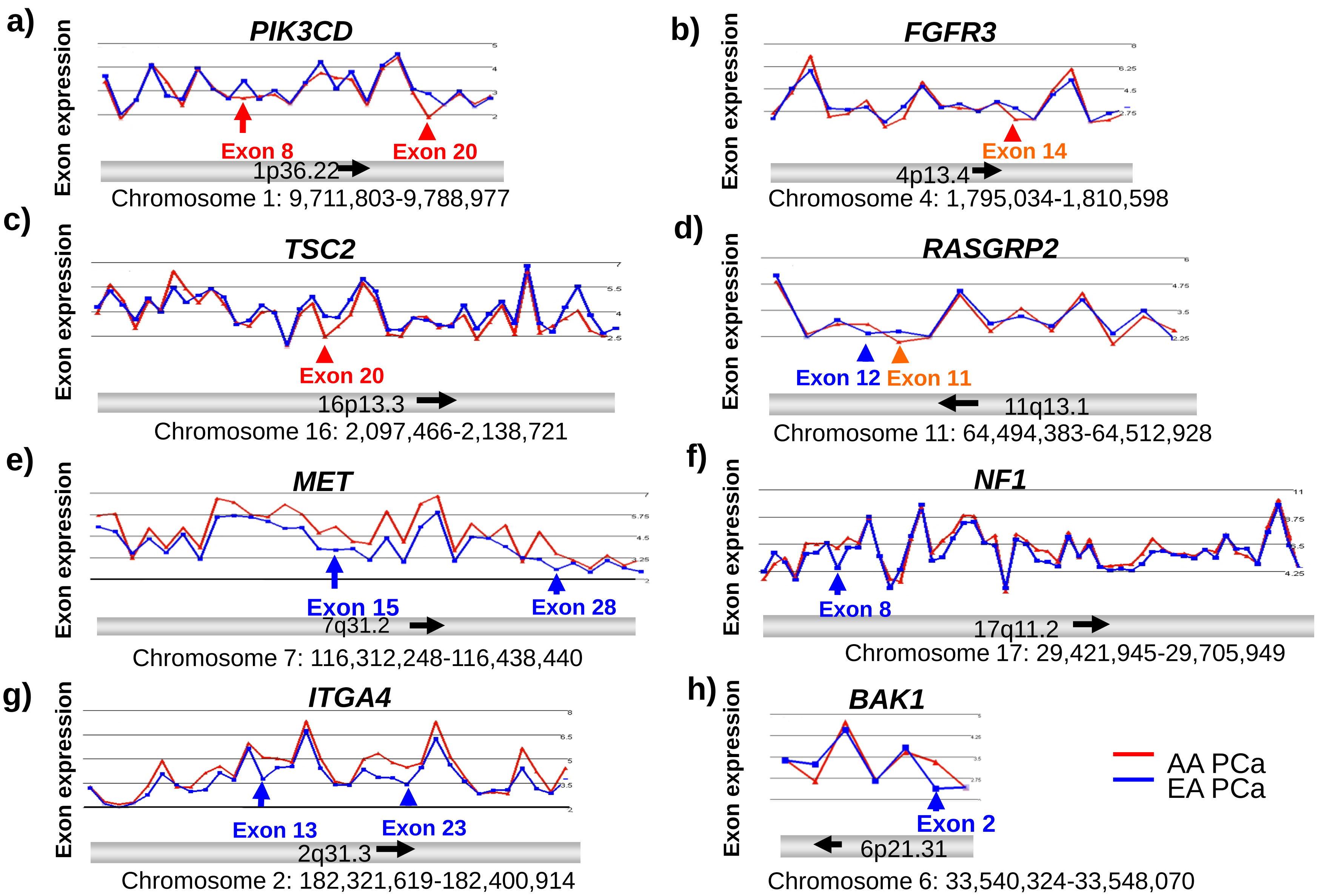

Supplementary Fig. 1. Affymetrix exon array profiling and Alternative Splice ANOVA modelling identifies differential splicing events in AA PCa vs. EA PCa. Log2 expression plots of exons for the indicated genes in AA (red lines) and EA (blue lines) PCa specimens. Genomic location of each gene is represented below each plot and black arrow below the x-axis indicates the coding direction of gene. Red arrow indicates the skipped exon in AA PCa specimens, blue arrow indicates the skipped exon in EA PCa specimens.

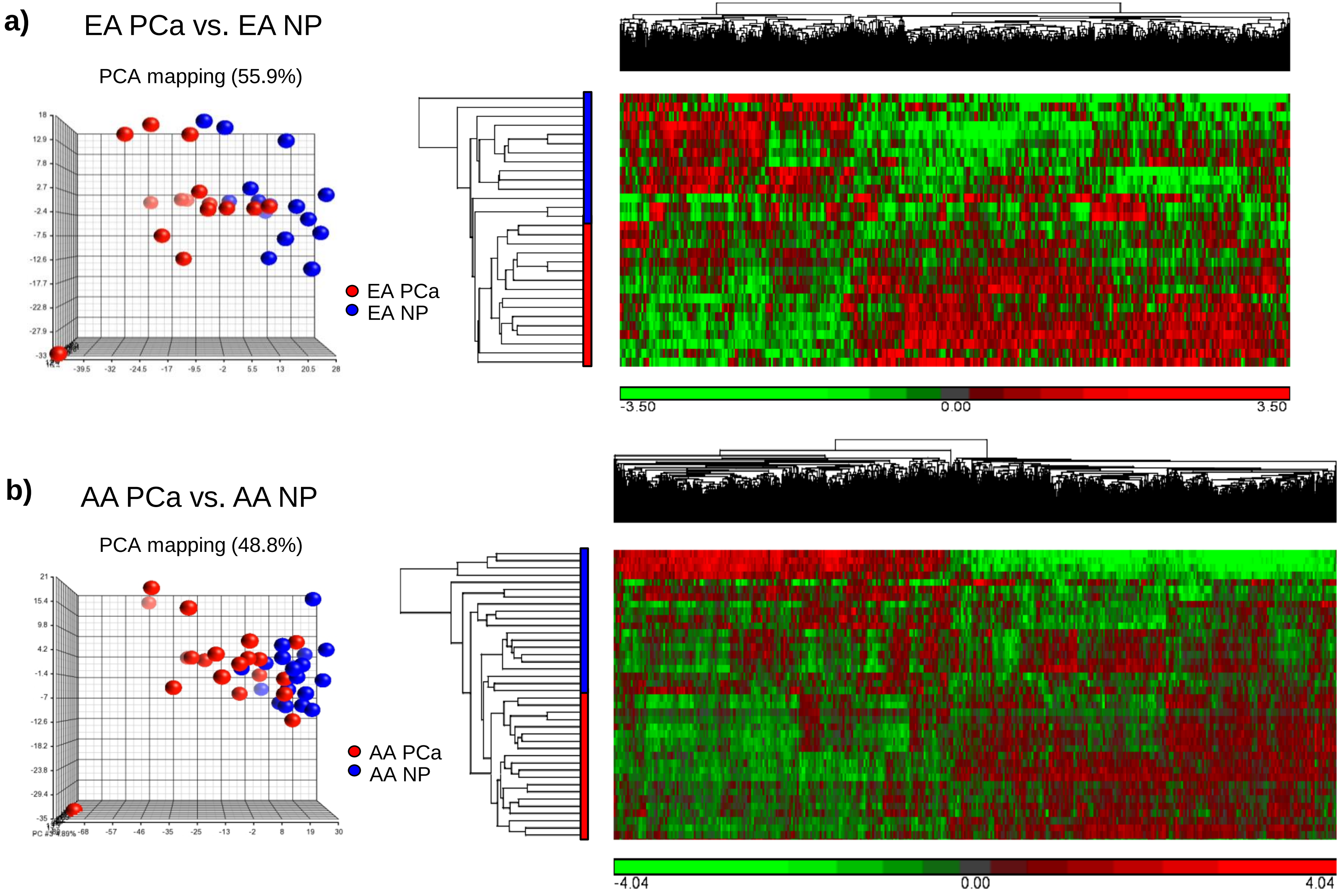

Supplementary Fig. 2. Heatmaps of alternative splicing differences in EA PCa vs. EA NP and AA PCa vs. AA NP specimens. (a) PCA plot and 2D-clustergram depicting 1,604 significant differentially expressed exons in 15 independent EA PCa vs. 15 independent EA NP specimens. (b) PCA plot and 2D-clustergram depicting 1,669 significant differentially expressed exons in 20 AA PCa vs. 20 AA NP specimens. PCa and NP specimens are represented by red and blue circles/bars, respectively. Rows represent specimens and columns represent exons in hierarchical clustergrams. Log2 expression values of exons were subjected to 2D hierarchical clustering using average linkage method and Euclidean distance.

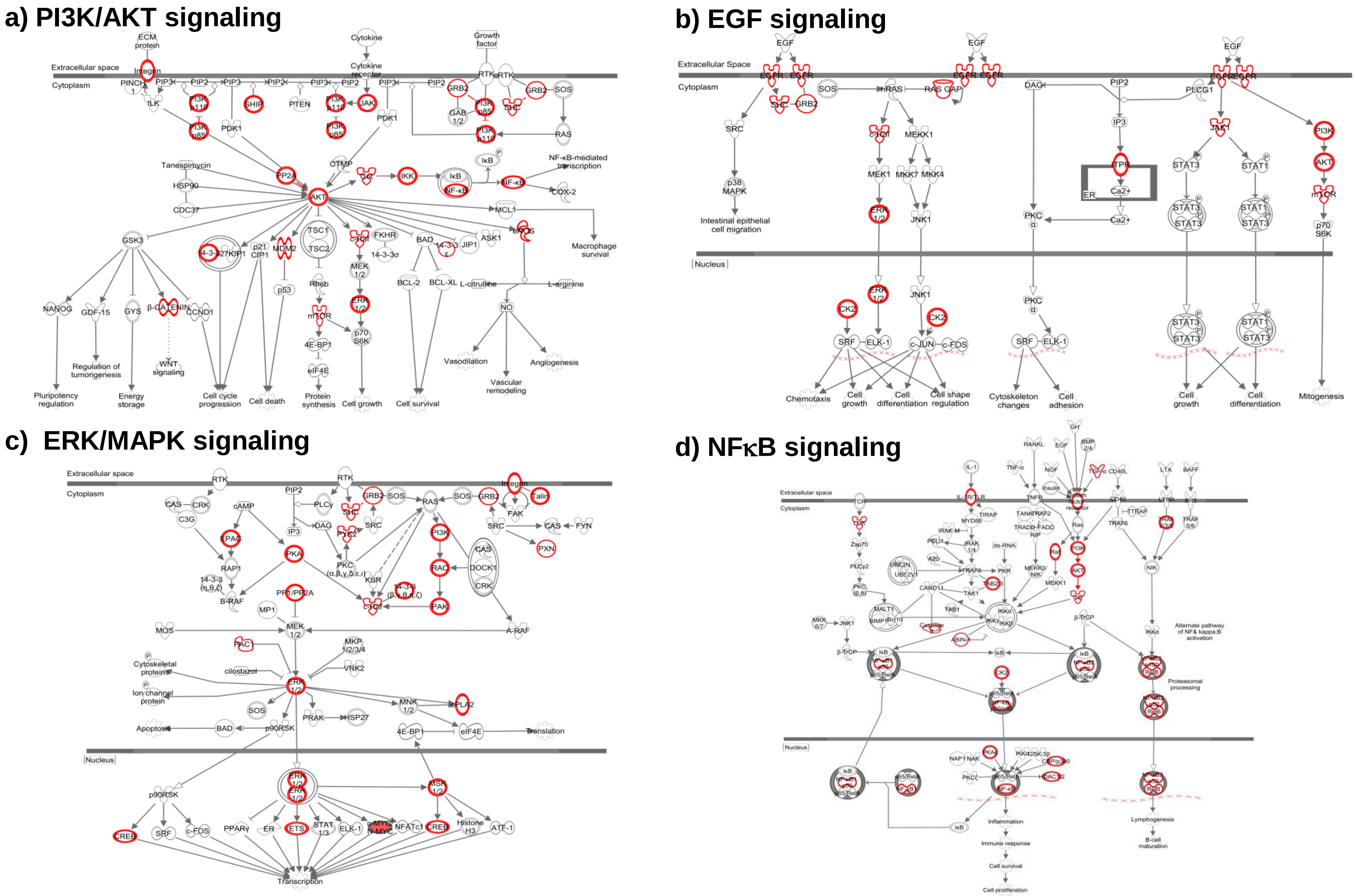

Supplementary Fig. 3. Oncogenic signaling pathways with an over-representation of differential splicing events in AA PCa vs. EA PCa. Pathways with statistical over-representation of genes exhibiting DS events (red outlined circles) were identified by a Fisher's exact test implemented using the Ingenuity Pathway Analysis (IPA) program.

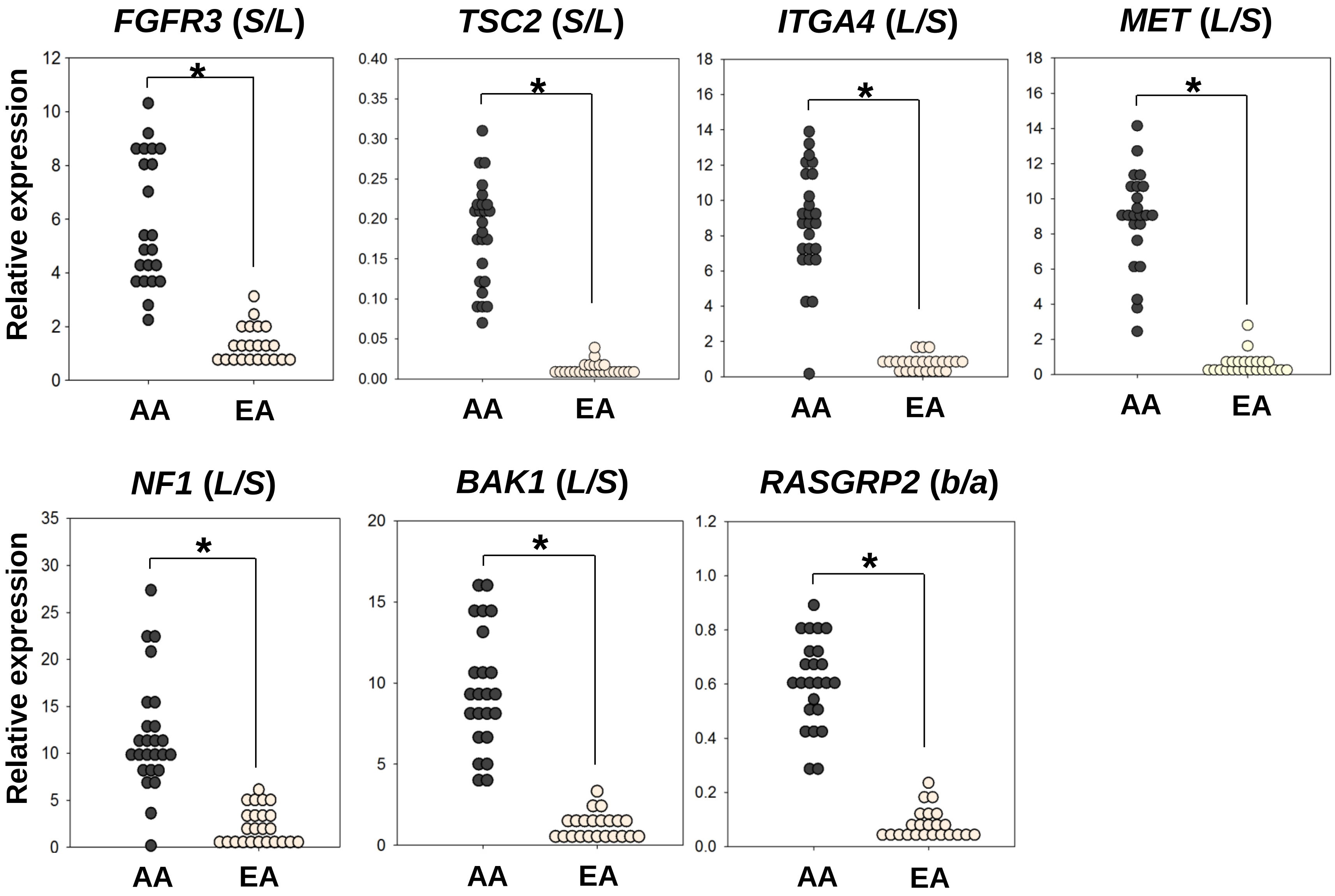

Supplementary Fig. 4. Quantification of QRT-PCR results of race-specific/-enriched oncogene and tumor suppressor gene variants in AA and EA PCa specimens. Quantitative real time RT-PCR was performed on samples depicted in Fig. 3b. RNA from n= 22-25 AA PCa and n= 21-24 EA PCa specimens were analysed. Shown are the plots for the AA-specific/-enriched variants *FGFR3-S, TSC2-S, ITGA4-L, MET-L, NF1-L, BAK1-L* and *RASGRP2-b*; and plots for the EA-specific/-enriched variants *FGFR3-L, TSC2-L, ITGA4-S, MET-S, NF1-S, BAK1-S and RASGRP2-a*. *EIF1AX* and *PPA1* transcripts served as internal normalization controls. * $P < 0.05$ using Student t-test.

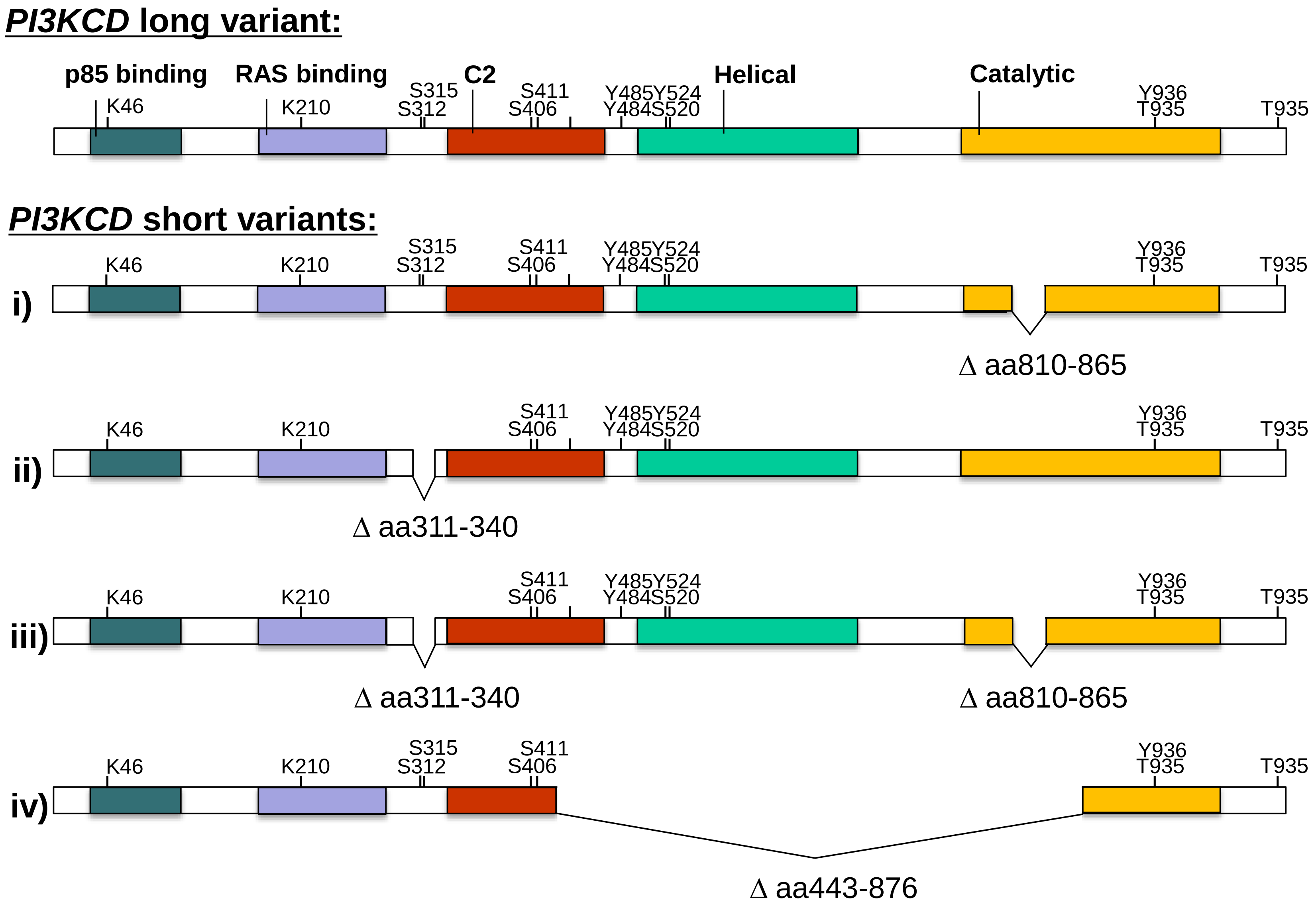

Supplementary Fig. 5. Molecular cloning of the long and short variants of *PI3KCD*. Schematic representation of the cloned *PI3KCD* variants. The long variant contains 24 exons based on sequence alignment to the *PI3KCD* genomic sequence described in the UCSC (genome.ucsc.edu/) or Ensembl Genome Browser (www.ensembl.org/). The short variants relative to the long variant are as follows: i) variant excluding exon 8 (encoding amino acids 311 to 340), variant excluding exon 20 (encoding amino acids 810 to 865), variant excluding both exons 8 and 20 and a large deletion variant excluding 1,299 nucleotides (encoding amino acids 443 to 876).

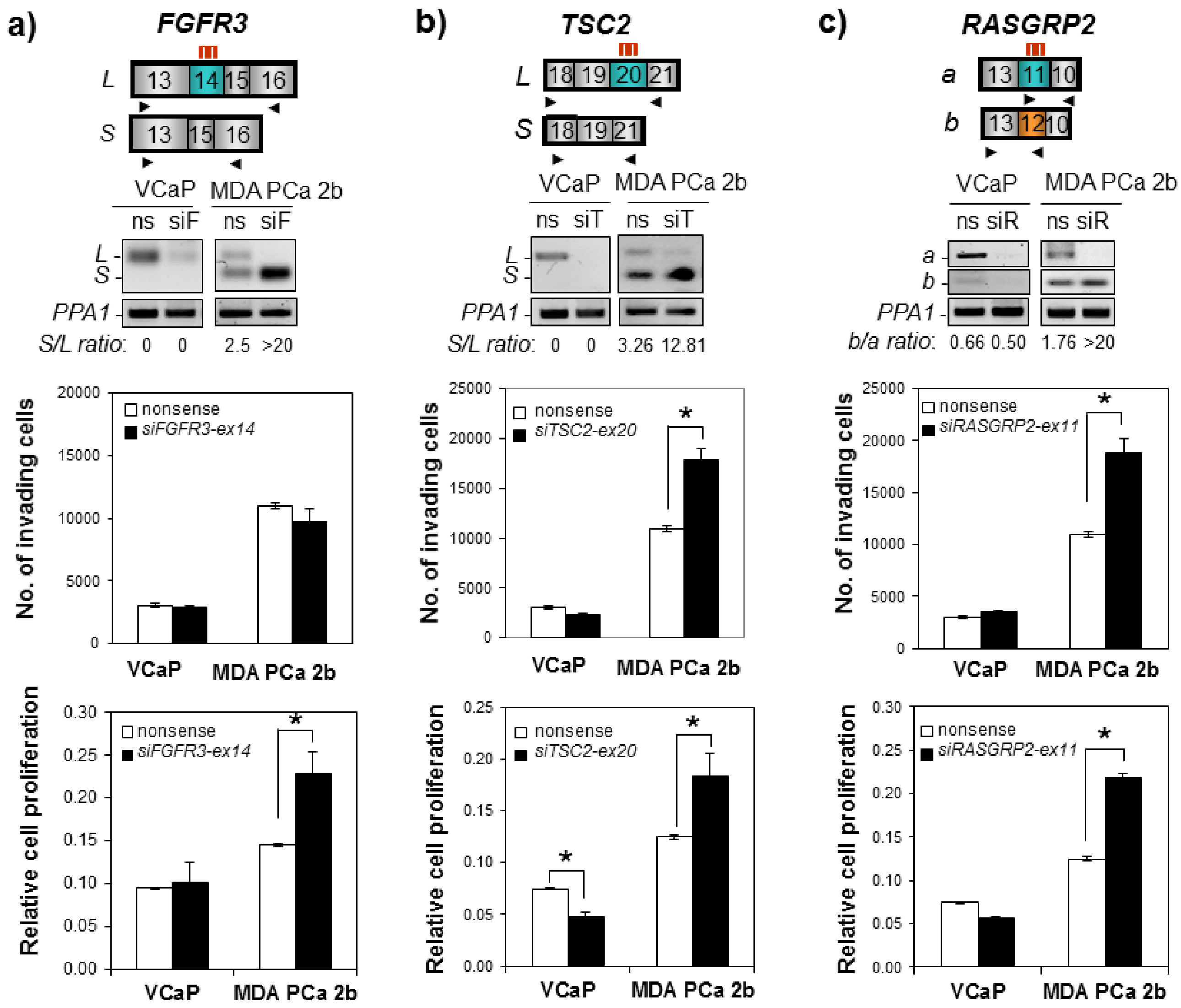

Supplementary Fig. 6. Enrichment of AA-specific/-enriched variants of *FGFR3*, *TSC2* or *RASGRP2* enhances proliferation and/or invasion of AA PCa cell line MDA PCa 2b. (a) Exon 14-specific siRNA (siF) was designed to target the EA- enriched *FGFR3-L* variant. (b) Exon 20-specific siRNA (siT) was designed to target the EA-enriched *TSC2-L* variant. (c) Exon 11-specific siRNA (siR) was designed to target the race-independent *RASGRP2-a* variant. Knockdown efficiency was determined by the variant ratio *(-S/-L* or *-b/-a* ratio) derived from the RT-PCR reactions in EA PCa cell line VCaP and AA PCa cell line MDA PCa 2b (upper panels). RT-PCR representative images of n = 3-5 independent knockdown experiments. Enrichment of the AA-specific/-enriched *FGFR3-S*, *TSC2-S* or *RASGRP2-b* variants augmented proliferation and/or invasion of AA PCa cell line MDA PCa 2b (bottom panels). Data presented as the mean ± SEM of n=3-5 independent experiments for each treatment group. *$P < 0.05$ by ANOVA with Tukey’s post-hoc test.

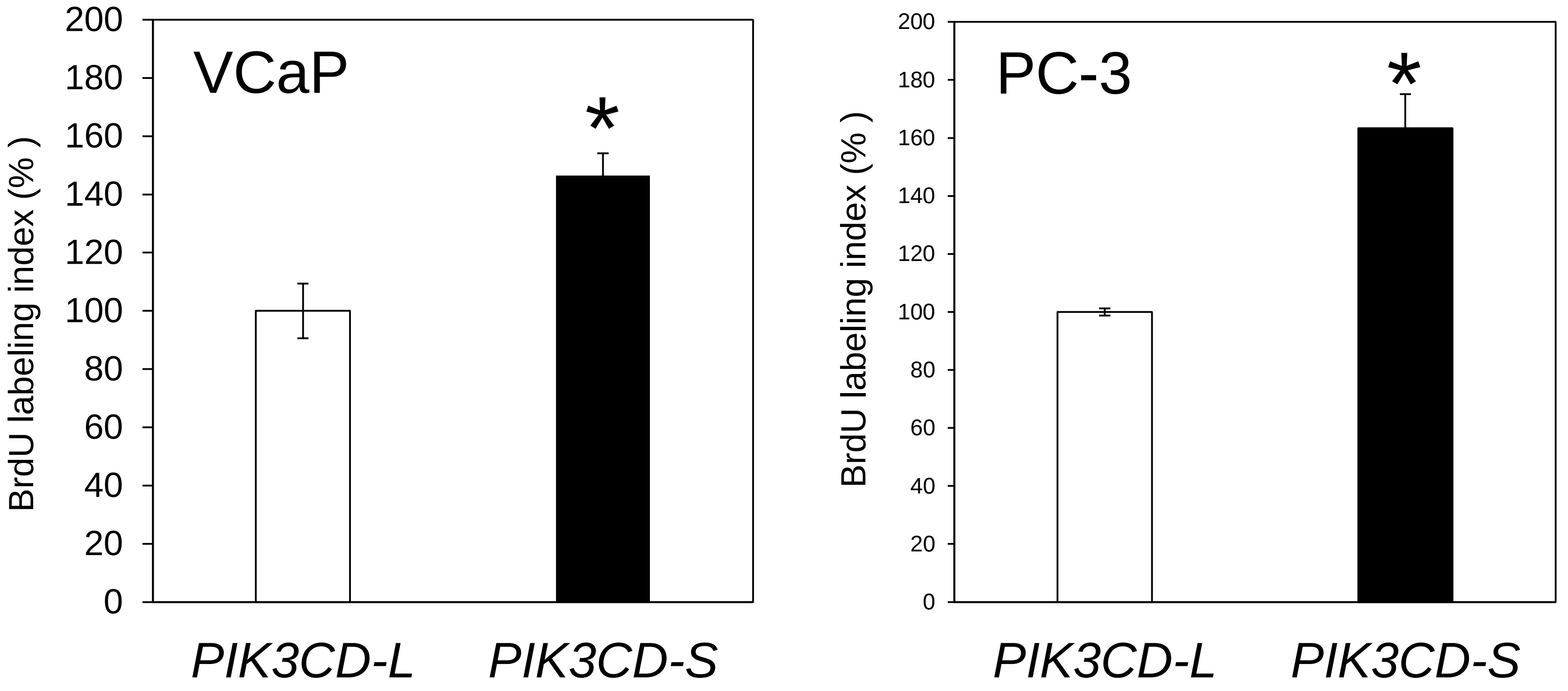

Supplementary Fig. 7. Baseline proliferative activity (BrdU labeling) of PC-3 and VCaP cell over-expressing PI3Kδ-S and PI3Kδ-L. Proliferation was assessed using a BrdU labeling assay. Data presented as the mean ± SEM of n=3-6 independent experiments for each treatment group. *$P < 0.05$ by ANOVA with Dunnett's post-hoc test.

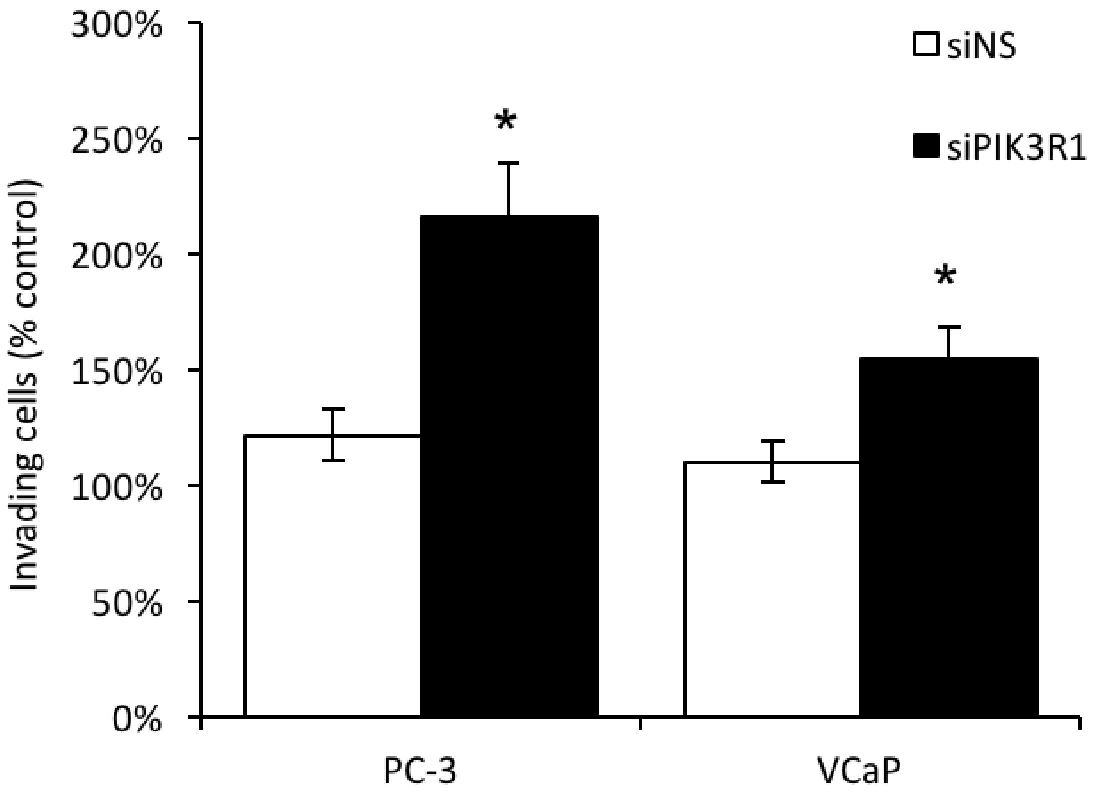

Supplementary Fig. 8. Knockdown of p85α increases invasive activity of EA PCa cell lines. Invasion was assessed using Matrigel assay following siRNA-mediated knockdown of p85a (PIK3R1). SiRNA-SMART pool for targeting *PIK3R1* was purchased from Dharmacon (Lafayette, CO). Data presented as the mean ± SEM of n=3-4 independent experiments for each treatment group. *P < 0.05 by ANOVA with Dunnett's post-hoc test. siNS, Nonsense siRNA control.

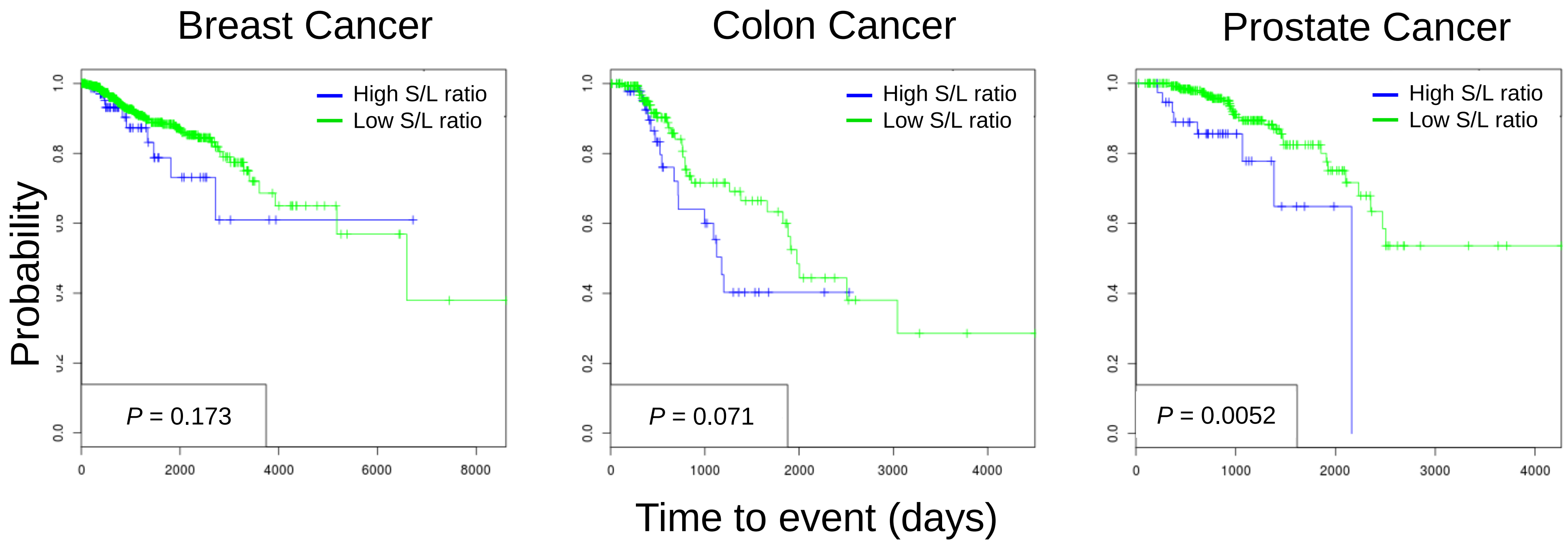

Supplementary Fig. 9. Survival plots for breast, colon and prostate cancer patients with high and low PIK3CD-S/PIK3CD-L expression ratios. RNA-Seq and disease free survival data for breast (n = 1,068 patients), colon (n = 277 patients) and prostate cancers (n = 494 patients) were obtained from The Cancer Genome Atlas (TCGA) (https://tcga-data.nci.nih.gov/tcga/). *P*-values for survival curves were determined by the log-rank test.

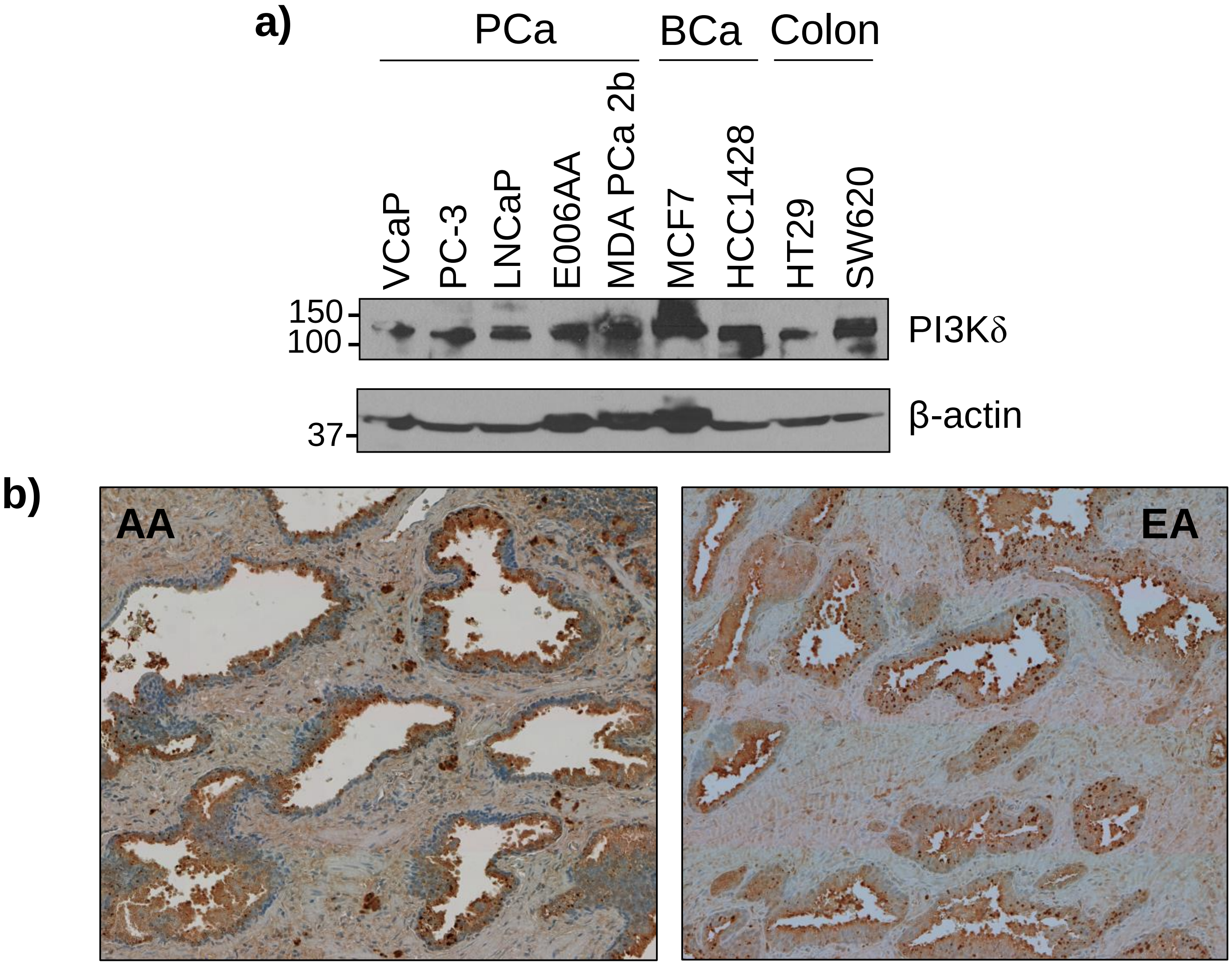

Supplementary Fig. 10. PI3Kδ expression in patient specimens and PCa cell lines. (a) Western blot analysis of cell lysates from PCa cell lines (VCaP, PC-3, LNCaP, E006AA and MDA PCa 2b), breast cancer (BCa) cell lines (MCF7 and HCC1428) and colon cancer cell lines (HT29 and SW620). β-actin served as loading control. Representative images from n = 3-4 independent western blot experiments. (b) Immunohistochemistry (IHC) analysis of PI3Kδ expression in formalin-fixed, paraffin-embedded (FFPE) tissue samples derived from AA and EA PCa patients. Representative images from n = 3 independent IHC assays.

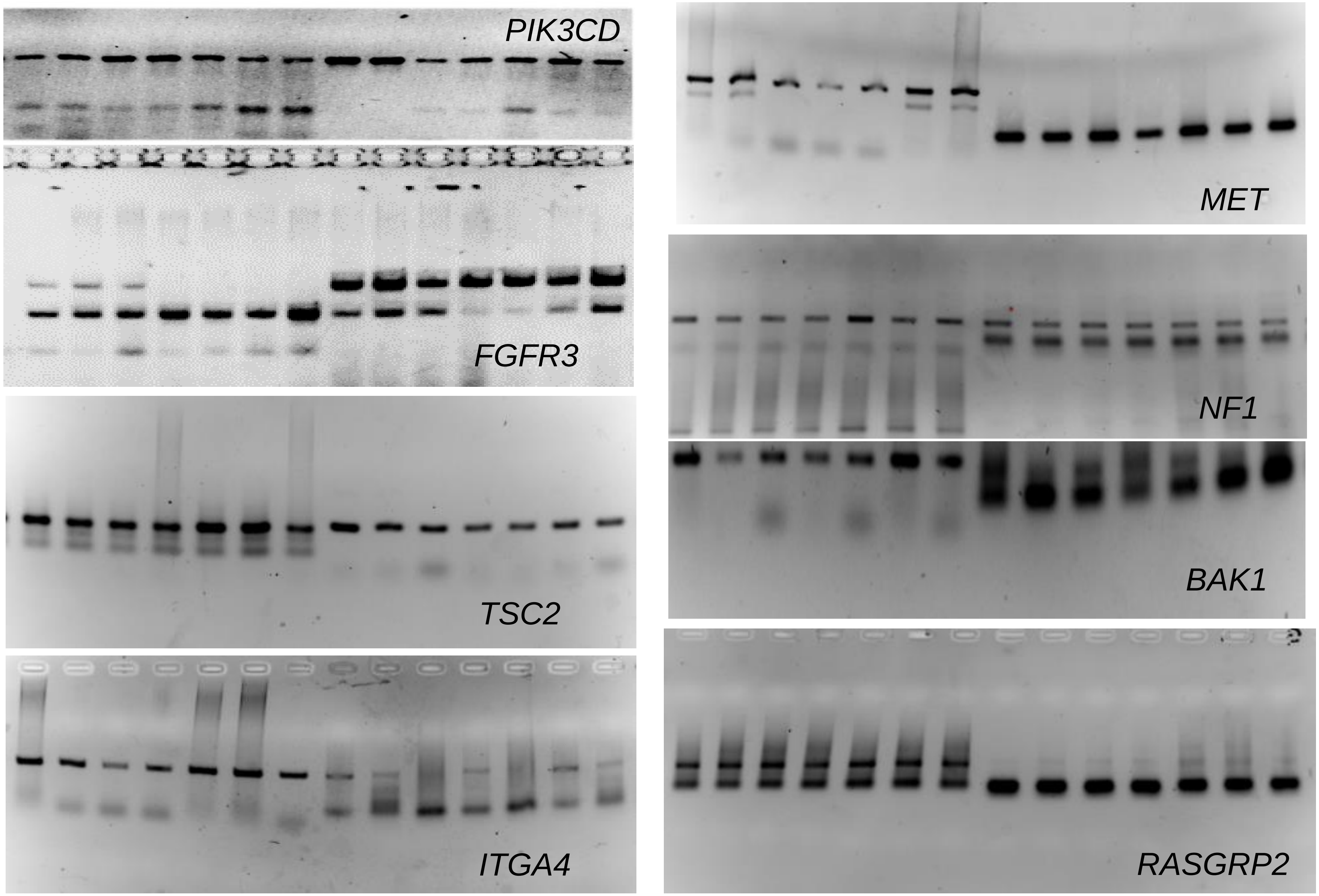

Supplementary Fig. 11. Unprocessed gel images for the RT-PCR results in Fig 3b.

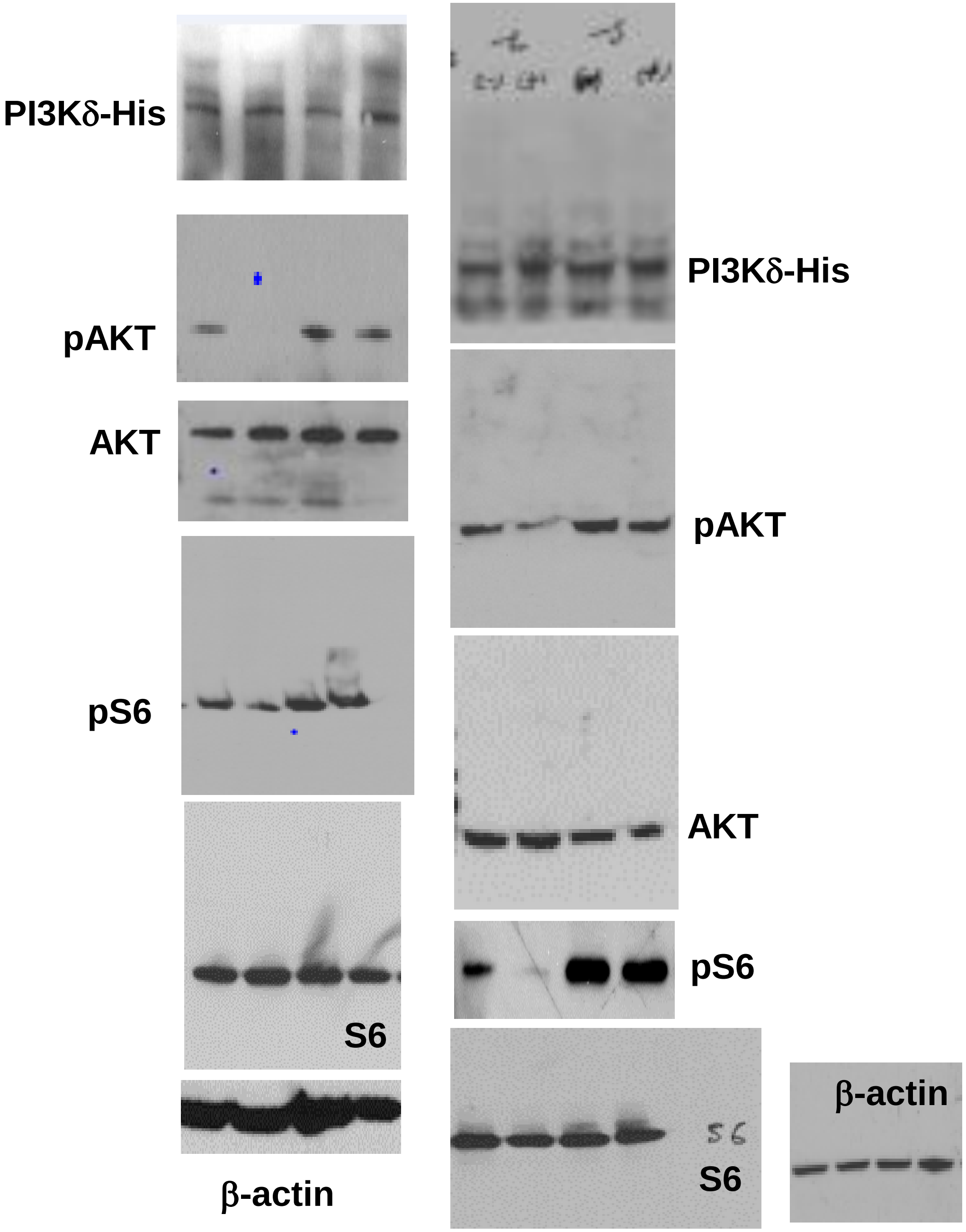

Supplementary Fig. 12. Unprocessed blot images for the western results in Fig 5a.

Fig. 7b

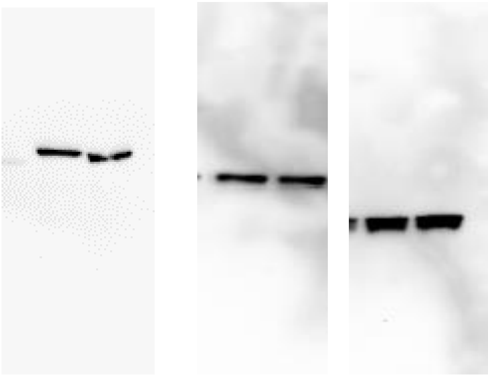

Fig. 7b

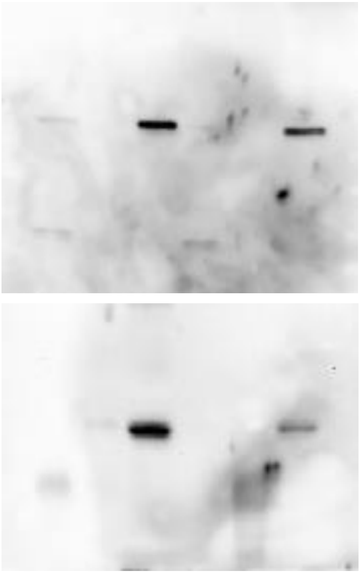

Fig. 7d

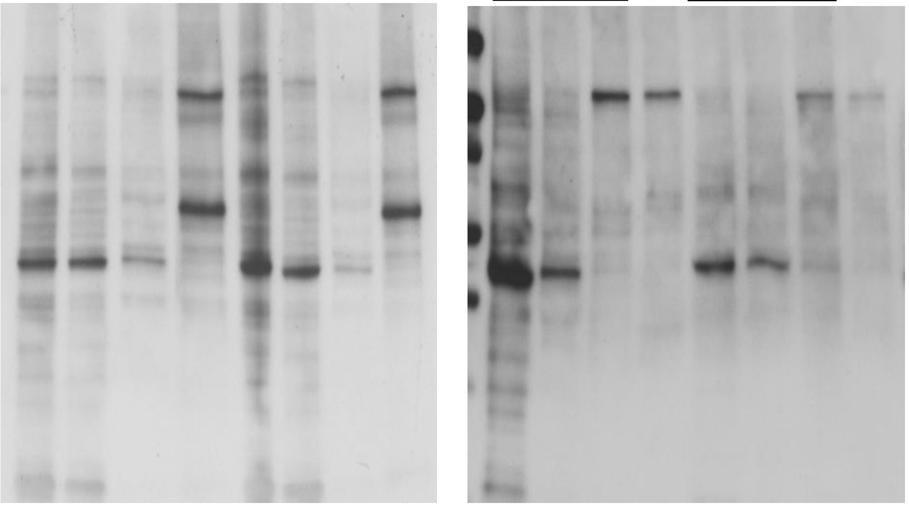

Fig. 7d

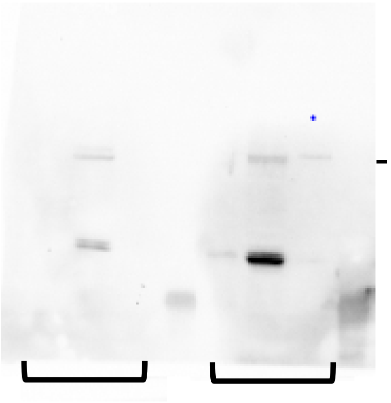

Supplementary Fig. 13. Unprocessed blot images for the western results in Fig 7.
